# Ecological and social factors influence interspecific pathogens occurrence among bees

**DOI:** 10.1038/s41598-024-55718-x

**Published:** 2024-03-01

**Authors:** Rossella Tiritelli, Simone Flaminio, Laura Zavatta, Rosa Ranalli, Manuela Giovanetti, Donato Antonio Grasso, Stefano Leonardi, Marta Bonforte, Chiara Benedetta Boni, Elena Cargnus, Roberto Catania, Francesca Coppola, Marco Di Santo, Michelina Pusceddu, Marino Quaranta, Laura Bortolotti, Antonio Nanetti, Giovanni Cilia

**Affiliations:** 1 CREA Research Centre for Agriculture and Environment (CREA-AA), Via di Corticella 133, 40128 Bologna, Italy; 2 Department of Chemistry, Life Sciences and Environmental Sustainability, University of Parma, Parco Area delle Scienze 11/A, 43124 Parma, Italy; 3 Laboratory of Zoology, Research Institute for Biosciences, University of Mons, Av. Champ de Mars 6, 7000 Mons, Belgium; 4 Departement of Agriculture and Food Sciences, University of Bologna, Via Giuseppe Fanin 42, 40127 Bologna, Italy; 5 ZooPlantLab, Department of Biotecnology and Biosciences, University of Milano-Bicocca, Piazza dell’Ateneo Nuovo 1, 20126 Milan, Italy; 6 Department of Agriculture, Food and Environment, University of Catania, Via Santa Sofia 100, 95123 Catania, Italy; 7 Department of Veterinary Sciences, University of Pisa, Viale Delle Piagge 2, 56124 Pisa, Italy; 8 Department of Agricultural, Food, Environmental and Animal Sciences, University of Udine, Via Delle Scienze 206, 31000 Udine, Italy; 9 Maiella National Park, Via Badia 28, 67039 Sulmona, Italy; 10 Department of Agricultural Sciences, University of Sassari, Viale Italia 39A, 07100 Sassari, Italy; 11 National Biodiversity Future Center (NBFC), Piazza Marina 61, 90133 Palermo, Italy

**Keywords:** Pollinators, Agricultural land use, Epidemiology, Bee functional traits, Disease transmission, Spillover, RNA, Environmental impact, Microbiology, Molecular biology, Entomology

## Abstract

The interspecific transmission of pathogens can occur frequently in the environment. Among wild bees, the main spillover cases are caused by pathogens associated with *Apis mellifera*, whose colonies can act as reservoirs. Due to the limited availability of data in Italy, it is challenging to accurately assess the impact and implications of this phenomenon on the wild bee populations. In this study, a total of 3372 bees were sampled from 11 Italian regions within the BeeNet project, evaluating the prevalence and the abundance of the major honey bee pathogens (DWV, BQCV, ABPV, CBPV, KBV, *Nosema ceranae*, *Ascosphaera apis*, *Crithidia mellificae*, *Lotmaria passim*, *Crithidia bombi*). The 68.4% of samples were positive for at least one pathogen. DWV, BQCV, *N. ceranae* and CBPV showed the highest prevalence and abundance values, confirming them as the most prevalent pathogens spread in the environment. For these pathogens, *Andrena, Bombus, Eucera* and *Seladonia* showed the highest mean prevalence and abundance values. Generally, time trends showed a prevalence and abundance decrease from April to July. In order to predict the risk of infection among wild bees, statistical models were developed. A low influence of apiary density on pathogen occurrence was observed, while meteorological conditions and agricultural management showed a greater impact on pathogen persistence in the environment. Social and biological traits of wild bees also contributed to defining a higher risk of infection for bivoltine, communal, mining and oligolectic bees. Out of all the samples tested, 40.5% were co-infected with two or more pathogens. In some cases, individuals were simultaneously infected with up to five different pathogens. It is essential to increase knowledge about the transmission of pathogens among wild bees to understand dynamics, impact and effects on pollinator populations. Implementing concrete plans for the conservation of wild bee species is important to ensure the health of wild and human-managed bees within a One-Health perspective.

## Introduction

With their pollination service, bees contribute to maintaining the health and function of the ecosystems, ensuring the biodiversity and reproduction of plants and guaranteeing crop production and food security^1–4^. Although the demand for pollination services is increasing^5,6^, many reports are revealing declines in bee diversity worldwide, a reduction of geographic ranges for several species, extinction for some species and a decrease in local abundance^3,7–11^. Several factors are contributing to this decline, such as pathogens spread, pesticides, climate change and habitat loss^3,7,12,13^. Regarding pathogens spread, the diffusion and introduction of new diseases (EIDs, Emerging Infectious Diseases) can happen due to host shifts of pathogens between populations^3,12,14^. The risk of infection increases where human activities occur, like commercial management of bees, mass breeding, transport and trade beyond their original ranges^3,15,16^. Different correlational evidence hypothesized that the most worldwide managed *Apis mellifera* can act as a source of pathogens (maintenance host), which can spread into wild bee species (incidental host)^17–22^. However, this hypothesis includes historical reasons, since many pathogens and diseases were originally discovered in honey bees^18,21,23^. In addition, the route of interspecific transmission is difficult to determine and in most cases is unknown^23^. Pathogen spread is promoted when infected bees contaminate the same environment, that is populated by other new host species^21^. This indirect transmission can occur through sharing of food, fecal contact, contact with another infected organism, a vector and predation^18,23–36^. Sharing the same contaminated resources, such as flowers, pollen, honey and nectar, is a successful route of infection^23^. Flowers are described as “dirty doorknobs”, as they facilitate the spread of pathogens and can contain infective particles deposited by infected hosts^25–29^. Since bees are obligate flower visitors, the distribution, diversity, and abundance of floral resources in the environment are important for promoting interspecific interactions and potential pathogen diffusion^37^. Expansion of agriculture and landscape simplification can affect the floral availability for bees and impact the dynamics and transmission of pathogens^38,39^. Furthermore, meteorological conditions can alter both lifestyles of the host and pathogen persistence in the environment^20^; for example, in the case of viruses, high levels of UV can rapidly deactivate virus particles and the same happens when temperatures increase^40,41^. Interactions between bees and the environment are mediated by the biological traits of bees, affecting disease dynamics, susceptibility to infection and exposure to pathogens^37,42^. Yet, only a few studies have recently begun to investigate functional trait role in influencing the prevalence of pathogens^37,42^. Sociality could exacerbate or mitigate the diffusion of disease because living in a colony implies both positive and negative aspects in terms of hygiene^37,42–45^. Different nesting locations can influence pathogen transmission and their persistence in the nest^42,46,47^. Additionally, diet and preference for flower resources can modulate the spread of pathogens, since specialist bees collect pollen on a limited number of plant species, while generalist bees forage on multiple plants^29,37,38,42,48^. Also, voltinism may influence the diffusion of diseases, through the number of individuals generated and the nest density^49^. According to a One-Health approach, these traits, along with meteorological and environmental ones, could be crucial to understand pathogens dynamics between wild bees, in order to reduce the spread of diseases and promote concrete and effective conservation projects^50–52^. The purpose of this study was to investigate the occurrence and circulation of honey bee pathogens in the wild bees of Italy. Also, we aimed to use statistical models to assess the relative importance of factors influencing pathogen occurrence and to predict the risk of infection in wild bees. Accordingly, prevalence and abundance data were tested with apiary density, meteorological variables, agricultural management, and biological traits of bees.

## Material and methods

### Sampling

**Figure 1:**
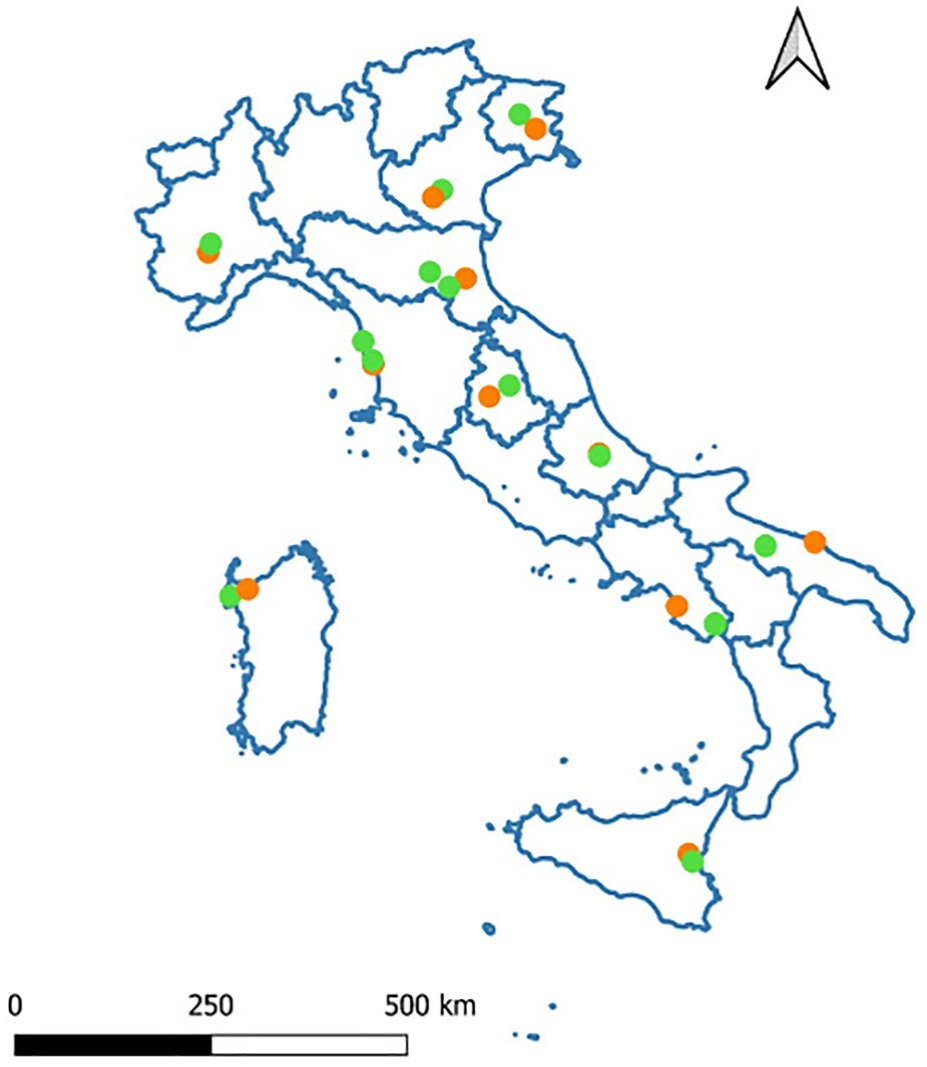
The geographical location of the sampling agricultural site. Orange spots represent intensive agricultural sites, while the green spots represent semi-natural agricultural sites.

This study was conducted within the project BeeNet, which monitors honey bee colonies and wild bees in Italy in managed agricultural areas on two Corine Land Cover Categories: intensive (category 2.1.1.1) and semi-natural (category 2.4.3)^53^. The sampling was conducted once a month, from February to September 2022, in 24 sites located in 11 regions of Italy (Fig. 1). In each site, all sampling was carried out excluding fields blooming and focusing on spontaneous vegetation likewise field margins, ditches and meadows^21^. Table S1 reports the sampling site acronym (used across the text), the agro-environmental characteristics (intensive, semi-natural) and geographic reference (region, closest town, province) of each sampling site.

The sampling was conducted on sunny and non-windy days, with an average temperature above 15 °C^21,42^. Sampling consisted in collecting wild bees foraging on flowers and flying. Honey bees were caught up to a maximum of 5 individuals per sampling, given their abundance. Bees were collected using the sweep net technique for one effective hour and each individual was placed in a sterile single 2-ml microtube or 15-ml tube^21^. After collection, the tubes were placed in a cooler bag with frozen packs to maintain samples at low temperature. Upon arrival at the CREA laboratories in Bologna, the specimens were all identified to the species level^21,42^.

Before being identified all collected bees were placed at −80 °C for 30 min. Identification was performed under a stereomicroscope, with the individual placed in a Styrofoam container with dry ice to prevent RNA degradation. Following identification, samples were kept at −80 °C until analysis.

### Extraction of nucleic acids

All samples were washed in 95% ethanol before extraction to eliminate any external microbiological contaminations. Each bee was examined individually. The sample was put in a 2-ml microtube with 500 µl of DNA/RNA Shield (Zymo Research, Irvine, CA, USA) and crushed for 3 min at 30 Hz with a TissueLyser II (Qiagen, Hilden, Germany), as previously reported^21,54,55^. The suspensions were separated into two aliquots from which DNA and RNA were extracted separately. The extraction of the DNA and RNA was performed using a Quick DNA Microprep Plus Kit (Zymo Research) and a Quick RNA Microprep Plus Kit (Zymo Research), respectively, following the modified manufacturer's instructions for solid tissue processing^21,35,56^. The extracted nucleic acids were eluted in 200 µl of DNAase-RNase-free water and kept at −80 °C until the qPCR analysis.

### Quantitative real-time PCR (qPCR) assays

A quantitative Real-Time PCR (qPCR) analysis was performed to determine the abundance of each pathogen in the samples using the extracted DNA and RNA. *Ascosphaera apis*, *Nosema ceranae* and trypanosomatids (*Lotmaria passim, Crithidia mellificae* and *C. bombi*) were detected using DNA, while viruses (deformed wing virus—DWV; black queen cell virus—BQCV; chronic bee paralysis virus—CBPV; acute bee paralysis virus—ABPV; Kashmir bee virus—KBV) were investigated using RNA. The primers used for the qPCRs are reported in Table S2.

A total reaction volume of 10 µl was produced for each target gene using SYBR™ green assays with forward and reverse primers and nucleic acid extract adding 2 µl of extracted DNA or RNA, as reported in previous studies^21,57^. The SYBR PowerUp™ SYBR™ Green Master Mix (ThermoFisher, Waltham, MA, USA) and the Power SYBR™ Green Cells-to-CT™ Kit (ThermoFisher Scientific) were used for the DNA and RNA, respectively. The qPCRs were carried out using a QuantStudio™ 3 Real-Time PCR System (ThermoFisher Scientific), according to the protocols for each gene sequence^34,58–63^. DNA and RNA previously extracted from positive honey bees were employed as positive controls for each pathogen investigated; while, as a negative control, sterile water was used. All the analyses were carried out in duplicate.

A standard curve was created for each of the target genes by amplifying serially diluted recombinant plasmids containing the pathogen-specific DNA and RNA fragment from 1 × 10^1^ to 1 × 10^9^ copies in a qPCR assay on QuantStudio TM 3 Real-Time PCR System (ThermoFisher Scientific), as reported in previous studies using the amplification and quantification protocols^34,59–64^.

### Statistical analysis

For the statistical analysis, a database (Table S3) was created including all sampled bees (with associated identification code, species name, region, month and sampling site). Besides, regions were associated with a latitude-based macro category (North, Central, or South Italy), while sampling sites were also clustered according to environmental management (semi-natural or intensive). Two meteorological variables were selected for their possible influence on the prevalence and abundance of pathogens: maximum daily temperature (Tmax) and daily mean relative humidity (RH). For each sampling day, the two meteorological data were obtained from the NASA Langley Research Center (LaRC) POWER Project (https://power.larc.nasa.gov/). The honey bee apiary density per kilometre for each province related to the sampling site was also included in the database, collecting data from the National Database of Zootechnical Registry, section Beekeeping (BDN) (https://www.vetinfo.it/j6_statistiche/#/report-pbi/45). The data were employed at the provincial level since it represented the smallest available territorial unit in BDN about apiary density. Five functional traits of bees were selected as explanatory variables: sociality, voltinism, nesting habits, diet specialization and foraging range. These traits have already been hypothesized to influence the prevalence of pathogens^42^. Data were obtained from the “Wild Bees Functional Traits Database” (S. Roberts, unpublished, pers. comm.), constantly updated by numerous researchers. Each species was clustered in terms of bee group (honey bee, wild bee and cuckoo bees), sociality (highly eusocial, primitively eusocial, communal, solitary or cleptoparasite), nesting habits (managed apiary, mining, renter, carder, mason, and cleptoparasite bees), voltinism (univoltine, bivoltine and multivoltine), diet specialization (lecticism) reduced in only three categories (oligolectic or polylectic) and foraging range (as intertegular distance in mm)^65^. Cuckoo bees were maintained separated from “wild bees" due to their distinctive anatomy and biology features: they lack of pollen collecting structures, and it is almost impossible to be sure a female cuckoo laid her eggs in the nest of a single host female or at least host females belonging to the same species.

The prevalence of pathogens was calculated as the ration between the number of bees positive for pathogens and the total number of collected individuals. The pathogen abundance was calculated as the log10 transformed average of the results obtained from the two technical replicates. Explorative analyses were carried out through Spearman’s correlation between pathogens’ copy numbers, and the heatmaps creation for prevalence and abundance visualization for each investigated variable.

Time trend was explored through the analysis of variance. The distribution of pathogens’ abundance and prevalence were tested with a Shapiro–Wilk test in order to test their normality. Since normality was not satisfied to carry out parametric analyses, we employed Kruskal–Wallis with Dunn-test as post-doc to investigate differences.

Prevalence and abundance for each pathogen were fit with a generalized linear model (GLM) and linear mixed effects models (LMER). Due to the low number of infections (N < 50), KBV, *L. passim*, *C. bombi*, *A. apis* and *C. mellificae* were excluded from these analyses. In order to test meteorological, environmental and biological factors on the probability of infection for each pathogen, a logistic regression (GLM) was fit. The risk of infection was tested as a binomial response variable, where 1 specified the infection, and 0 described the absence of infection. Meteorological, environmental and biological factors were selected as predictors variables. Interactions were included in the two models to see their combined effect on pathogens’ prevalence. Attention was paid to the interaction between apiary density and environmental management since the pathogens investigated are closely related to the honey bee and different environmental management could affect pathogens’ prevalence. The interaction between temperature and relative humidity may influence the persistence and viability of the pathogen in the environment. The interaction between temperature and environmental management may affect the pathogens exposition among different environmental management. It was necessary to create two separate models for problems in aliased variables found in biological traits. The first model was tested with all meteorological and environmental variables and the explanatory variable “group of bees” (honey bees, cuckoo bees and wild bees). Model (GLM_M1_) was fit following this general formula (R syntax):

$$\begin{aligned} & Y \, \sim \, Apiary\;density + T\max + RH + Latitude \, \\ & \quad + Environmental\;Management + T\max : \, RH \\ & \quad + Apiary\;Density: \, Environmental\;Management \\ & \quad + T\max : \, Environmental\;Management + Group\;of\;Bees \\ \end{aligned}$$

The second model was tested with the same data, but honey bee and cuckoo bee were excluded (GLM_M2_), with this general formula (R syntax):

$$\begin{aligned} & Y \, \sim \, Apiary\;density \, + \, T\max \, + \, RH \, + \, Latitude \\ & \quad + Environmental \, \;Management \, + \, T\max \, : \, RH \, \\ & \quad + Apiary \, \;Density: \, Environmental\; \, Management \, \\ & \quad + T\max : \, Environmental\;Management + Sociality \\ & \quad + Voltinism + Nesting + Lecticism + Foraging\;Range \\ \end{aligned}$$

The relationship between pathogens’ abundance and environmental and biological factors was evaluated using linear mixed-effects models (LMER). In these models, sampling sites were chosen as random effects and other meteorological, environmental and biological variables as fixed effects. The same approach to alias data was conducted for these models. Two different models were created. The first model (LMER_M1_) was fit using meteorological, and environmental variables and the explanatory variable “group of bees”, following this formula (R syntax):

$$\begin{aligned} & Y \, \sim \, Apiary\; \, density \, + \, T\max \, + \, RH \, + \, Latitude \, \\ & \quad + \, Environmental\; \, Management \, + \, T\max \, : \, RH \, \\ & \quad + \, Apiary\; \, Density \, : \, Environmental\; \, Management \\ & \quad + \, T\max \, : \, Environmental\;Management \, \\ & \quad + \, Group\;of\;Bees \, + \, \left( {1|Sampling \, site} \right) \\ \end{aligned}$$

The second model was tested with the same data, but honey bee and cuckoo bee were excluded (LMER_M2_), with this general formula (R syntax):

$$\begin{aligned} & Y \, \sim \, Apiary\;density + T\max + RH + Latitude \, \\ & \quad + Environmental\;Management + T\max \, : \, RH \, \\ & \quad + Apiary\;Density: \, Environmental\;Management \, \\ & \quad + T\max \, : \, Environmental\;Management + Sociality \\ & \quad + Voltinism + Nesting + Lecticism \, \\ & \quad + Foraging\;Range \, + \, \left( {1|Sampling \, site} \right) \\ \end{aligned}$$

Significance for all models was determined by calculating the Type-II analysis of variance, with the sequent test post-hoc Tukey to find significance between factors.

Finally, a chord diagram was created to show co-infection between individuals belonging to the same genus.

The significative threshold was set at 0.05.

All the analyses were conducted in R 4.2.2 (r-project.org). Data manipulation, analysis and graphical representation were carried out with *agricolae, car*, *caret*, *circlize, corrgram, corrplot, dplyr, ggplot2, rstatix, and sjplot* packages^66–75^.

## Results

A total of 3372 bees were collected and analysed from the11 regions of Italy, involved in the project. A high number of samples was reported for Tuscany (N = 637), Emilia-Romagna (N = 508) and Campania (N = 430), followed by Sicily (N = 383), Piedmont (N = 361), Friuli-Venezia Giulia (N = 272), Apulia (N = 229), Umbria (N = 171), Sardinia (N = 160), Veneto (N = 119) and Abruzzo (N = 102).

The highest number of bees was sampled in July (N = 631), while the lowest in February (N = 45).

**Figure 2:**
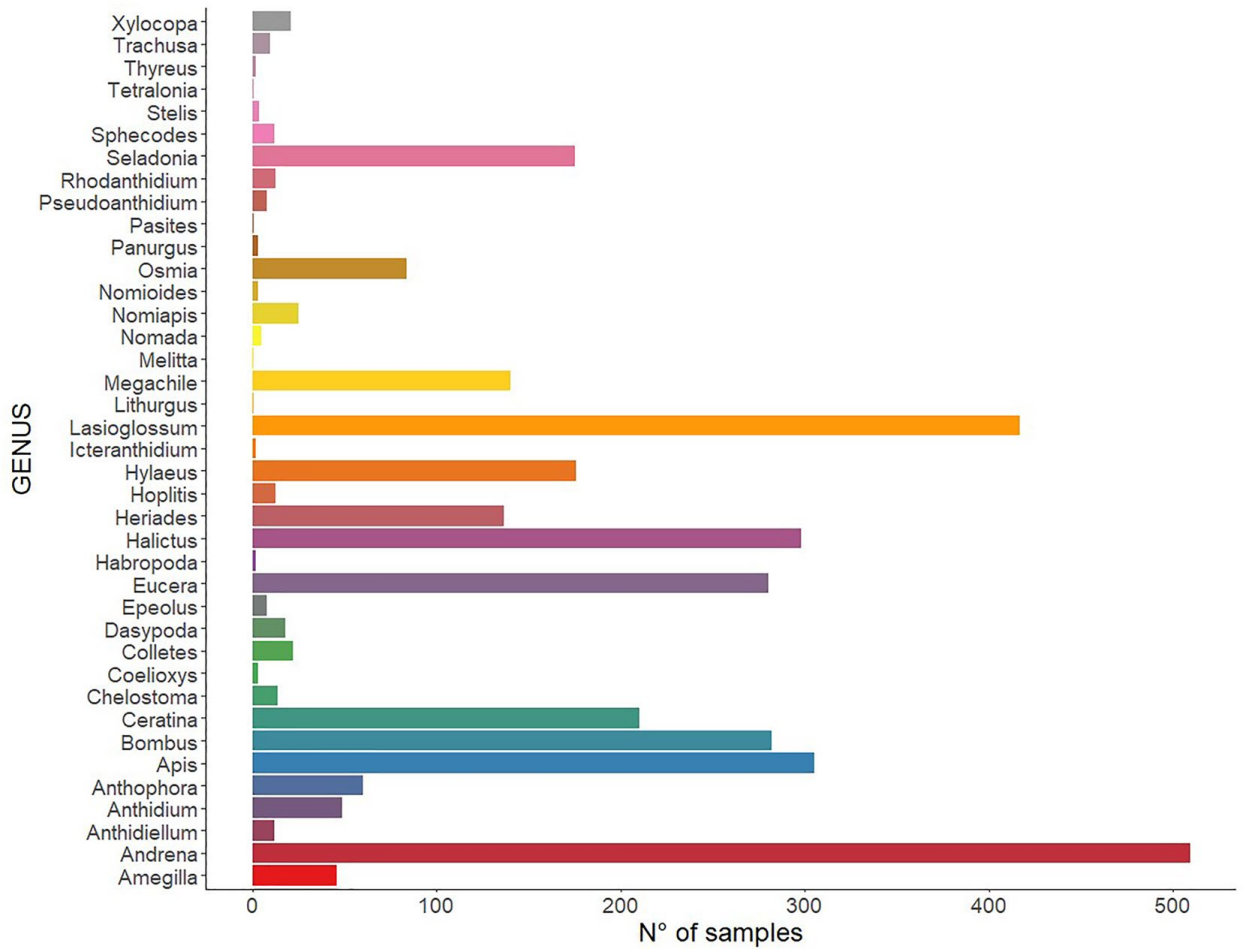
The total number of bees collected. Each genus is represented by different color.

Overall, 39 bee genera were identified (Fig. 2). The most frequently sampled genera were *Andrena* (Fabricius, 1775) (N = 509), *Lasioglossum* (Curtis, 1833) (N = 417), *Halictus* (Latreille, 1804) (N = 298), *Bombus* (Latreille, 1802) (N = 282), *Eucera* (Scopoli, 1770) (N = 280), *Ceratina* (Latreille, 1802) (N = 210), *Hylaeus* (Fabricius, 1793) (N = 176), *Seladonia* (Robertson, 1918) (N = 175), *Megachile* spp. (N = 140), *Heriades* spp. (N = 137), *Osmia* spp. (N = 84) and *Anthophora* (Latreille, 1802) (N = 60). Besides, a total of 305 *Apis mellifera* L. were collected. The number of samples for all other genera was under 50. The number of samples collected per region, sampling site and month are reported in Table S4.

The trypanosomatid *C. mellificae* was the only pathogen not detected in any samples. A total of 2307 of the individuals sampled were positive for at least one pathogen (68.4%) (Table 1). Overall, DWV, BQCV, *N. ceranae*, CBPV and ABPV were the five most prevalent pathogens. Lower prevalence was detected for *L. passim*, KBV, *C. bombi* and *A. apis*.

**Table 1:** The prevalence and the mean abundance (± standard deviation) for the investigated pathogens. Abundance value described the copy number of DNA or RNA pathogen.

Pathogen	Number of positive samples	Prevalence	Mean abundance	Standard deviation
*Ascosphaera apis*	6	0.2%	4.86 × 10^2^	3.96 × 10^4^
*Nosema ceranae*	767	22.7%	5.29 × 10^4^	7.21 × 10^5^
*Lotmaria passim*	41	1.2%	1.33 × 10^3^	2.78 × 10^4^
*Crithidia mellificae*	0	–	–	–
*Crithidia bombi*	20	0.6%	4.08 × 10^1^	1.53 × 10^2^
DWV	1738	51.5%	6.93 × 10^10^	7.82 × 10^11^
BQCV	896	26.6%	1.19 × 10^9^	2.76 × 10^10^
CBPV	538	16.0%	2.74 × 10^6^	2.38 × 10^8^
ABPV	409	12.1%	5.67 × 10^4^	3.47 × 10^3^
KBV	25	0.7%	6.01 × 10^1^	1.42 × 10^3^

DWV, deformed wing virus; BQCV, black queen cell virus; CBPV, chronic bee paralysis virus; ABPV, acute bee paralysis virus; KBV, Kashmir bee virus.

The pathogen with the highest abundance was DWV, followed by BQCV, CBPV, ABPV and *N. ceranae* (Table 1). All the other pathogens had an abundance below 10 × 10^2^. On average, the pathogen abundance for individuals sampled was found to be 7.05 × 10^9^ ± 2.45 × 10^11^.

The most prevalent pathogens among almost all genera were DWV, *N. ceranae* and BQCV (Fig. S1a). A high abundance of DWV was found in *Sphecodes* spp. (10 × 10^8^) and *Pasites* spp. (10 × 10^8^), while the abundance was high in *Lithurgus* spp. (10 × 10^5^) for *N. ceranae* and in *Tetralonia* spp. (10 × 10^5^) for BQCV. Regions of Abruzzo and Veneto showed a high prevalence of DWV (21.0% and 24.5%, respectively) (Fig. S1b). Sardinia showed a high abundance of DWV (10 × 10^7^) and BQCV (10 × 10^5^). A high prevalence of *N. ceranae* was detected for both Sardinia sampling site (SAES with 65.2% and SAAI with 61.5%) and a high abundance of DWV (SAES and SAAI with both 10 × 10^7^) (Fig. S1c.). Multivoltine bees showed a high prevalence and abundance of DWV (48.2% and 10 × 10^3^, respectively) (Fig. S1d). Prevalence and abundance data of multivoltine bees, highly eusocial bees (Fig. S1e) and the managed apiary (Fig. S1f.) corresponded perfectly since these categories were referred to *A. mellifera*. DWV prevalence and abundance were also high for bivoltine bees (68.3% and 10 × 10^5^, respectively) (Fig. S1d), cleptoparasite bees (60% and 10 × 10^5^, respectively) (Fig. S1e), communal bees (68.2% and 10 × 10^4^, respectively), excavator bees (57.4% and 10 × 10^4^, respectively) (Fig. S1f.) and oligolectic bees (59.6% and 10 × 10^4^, respectively) (Fig. S1g). DWV prevalence was reported as 80.0% in February and 18.8% in March and DWV abundance as 10 × 10^6^ in February and March (Fig. S1h).

### Seasonal trend

**Figure 3:**
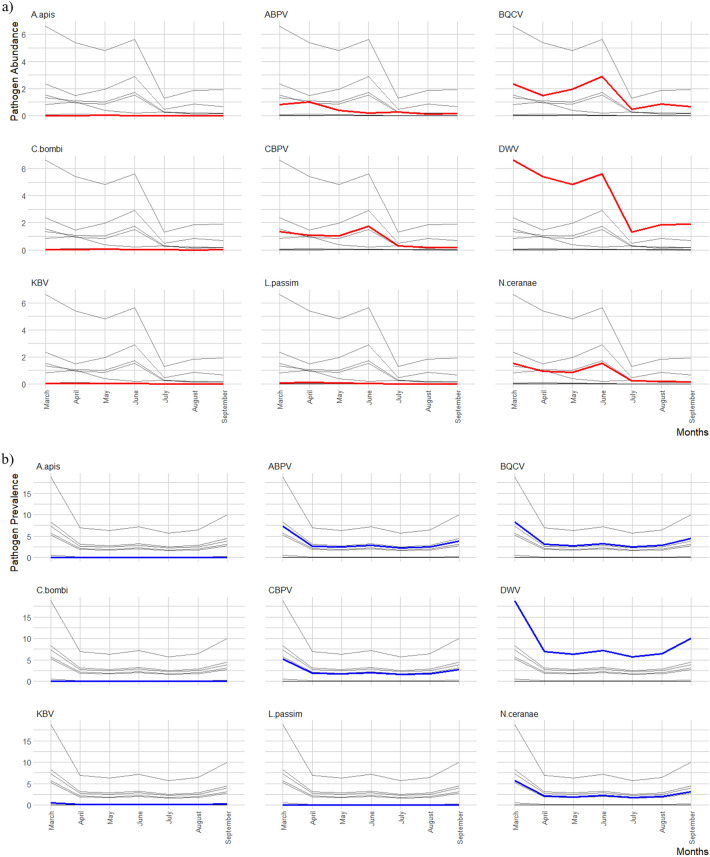
Pathogen abundance (**a**) and prevalence (**b**) throughout months (March–September). The time trend of each pathogen is highlighted in each graph. Abundance (red lines) is shown as a decimal logarithm and prevalence (blue lines) is shown as a percent.

A different seasonal trend considering pathogen abundance was noted (Fig. 3a). In March, a high abundance of DWV was detected. After a brief decline, DWV increased and reached another peak in June. Then, a drastic decline occurred from June to July and resumed increasing since September. A similar trend was found for BQCV and *N. ceranae*, but in these cases, the abundance decreased in September. In April a high abundance of ABPV was reported, while for CBPV the peak of abundance was reached in June. Significant differences among months were reported in Table S5. The abundance of *C. bombi* was significantly higher in April and August, while the abundance of *L. passim* was significantly higher in April. No significant differences were detected for KBV and *A. apis*.

The analysis of seasonal trend of pathogen prevalence throughout the months (Fig. 3b) started in March since the samples collected in February were only 45. In March, a high prevalence of the pathogens DWV, BQCV, ABPV, CBPV and *N. ceranae* was found. In April, the prevalence of these pathogens decreased drastically. A slight increase in prevalence was recorded in June. Then, pathogen prevalence again reached a peak in September. Significant differences among months were reported in Table S6. The prevalence of *C. bombi* was significantly higher in April and August, while the prevalence of *L. passim* was significantly higher in April. For KBV and *A. apis* no significant differences were detected.

### Pathogens’ correlation

**Figure 4:**
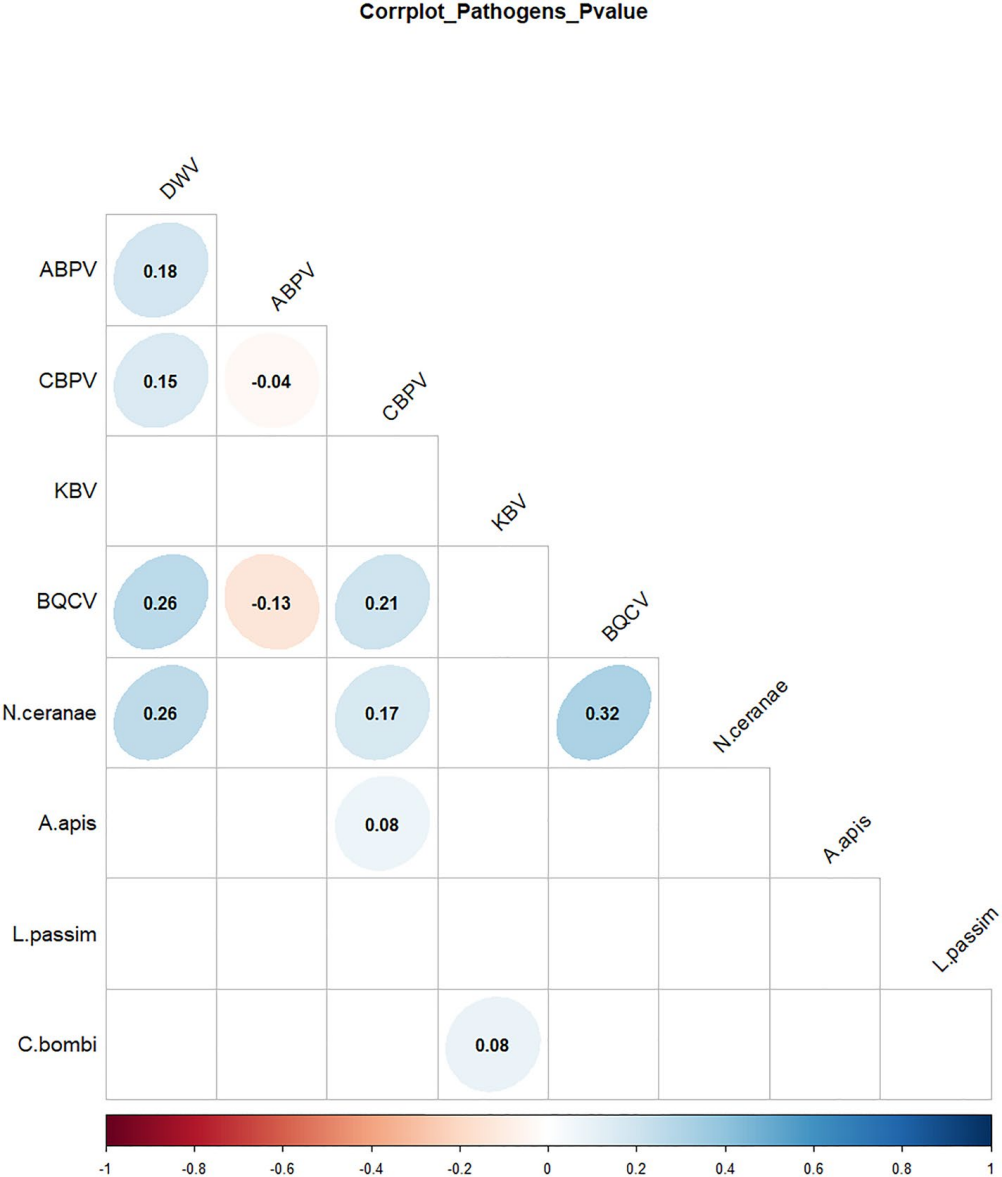
Positive and negative correlation between pathogens. Only significant correlations are shown. High or low values of correlation are shown with ellipsoidal or circular shapes, respectively.

A positive abundance correlation was detected between DWV and ABPV, DWV and CBPV, DWV and BQCV, and DWV and *N. ceranae*. For CBPV, a positive correlation was reported with BQCV, *N. ceranae* and *A. apis*. A positive correlation was found between KBV and *C. bombi* and between BQCV and *N. ceranae*. For ABPV, a negative correlation was detected with CBPV and BQCV (Fig. 4). Table S7 reported the p-value correlation between pathogens.

### GLM and LMER analysis

GLM are summarized in Table 2 and Figs. S2 and S3, while LMER results in Table 3 and Fig. S4 and S5. Post tests are available in Tables S8 and S9.

**Table 2:** Results of GLM_M1_ and GLM_M2_ fit for each analysed pathogen. Only significant values are shown.

Predictors		Response									
		DWV		ABPV		CBPV		BQCV		*N. ceranae*	
		GLM_M1_	GLM_M2_	GLM_M1_	GLM_M2_	GLM_M1_	GLM_M2_	GLM_M1_	GLM_M2_	GLM_M1_	GLM_M2_
Meteorological	T (Max)	< 0.001	< 0.001	< 0.001	< 0.001	< 0.001	< 0.001	–	–	< 0.001	< 0.001
	RH	< 0.001	< 0.001	< 0.001	–	= 0.001	= 0.002	< 0.001	< 0.001	< 0.001	< 0.001
	T (Max):RH	< 0.001	= 0.012	< 0.001	< 0.001	–	–	= 0.025	–	= 0.010	= 0.017
Environmental	Latitude	< 0.001	< 0.001	< 0.001	< 0.001	–	–	= 0.029	= 0.001	–	–
	Env. Man	–	–	< 0.001	< 0.001	–	= 0.012	< 0.001	< 0.001	–	–
	Apiary density	–	–	< 0.001	< 0.001	–	–	< 0.001	< 0.001	–	= 0.002
	Env. Man: Apiary Density	< 0.001	= 0.002	< 0.001	< 0.001	–	–	–	–	–	–
	T (Max): Env. Man	–	–	< 0.001	< 0.001	–	–	–	–	–	–
Bee functional traits	Bees	= 0.005	n.a	= 0.002	n.a	= 0.042	n.a	–	n.a	–	n.a
	Voltinism	n.a	< 0.001	n.a	= 0.005	n.a	= 0.004	n.a	< 0.001	n.a	< 0.001
	Sociality	n.a	< 0.001	n.a	= 0.003	n.a	= 0.039	n.a	< 0.001	n.a	< 0.001
	Nesting	n.a	< 0.001	n.a	–	n.a	–	n.a	< 0.001	n.a	< 0.001
	Lecticism	n.a	< 0.001	n.a	–	n.a	–	n.a	< 0.001	n.a	= 0.001
	Foraging range	n.a	< 0.001	n.a	–	n.a	–	n.a	< 0.001	n.a	–

Legend. n.a.: not available value (not tested variable); -: non-significant p-value.

**Table 3:** Results of LMER_M1_ and LMER_M2_ fit for each analysed pathogen. Only significant values are shown.

Predictors		Response									
		DWV		ABPV		CBPV		BQCV		*N. ceranae*	
		LMER_M1_	LMER_M2_	LMER_M1_	LMER_M2_	LMER_M1_	LMER_M2_	LMER_M1_	LMER_M2_	LMER_M1_	LMER_M2_
Meteorological	T (Max)	< 0.001	< 0.001	< 0.001	< 0.001	< 0.001	< 0.001	< 0.001	= 0.003	< 0.001	< 0.001
	RH	< 0.001	< 0.001	< 0.001	< 0.001	< 0.001	< 0.001	–	–	< 0.001	< 0.001
	T (Max):RH	–	–	= 0.014	< 0.001	–	–	–	–	–	–
Environmental	Latitude	–	–	–	–	–	–	–	–	–	–
	Env. Man	–	–	–	–	–	–	–	–	–	–
	Apiary density	–	–	–	–	–	–	–	–	–	–
	Env. Man: Apiary Density	–	–	–	–	–	–	–	–	–	–
	T (Max): Env. Man	–	–	< 0.001	< 0.001	–	–	< 0.001	< 0.001	–	–
Bee functional traits	Bees	< 0.001	n.a	< 0.001	n.a	= 0.029	n.a	–	n.a	< 0.001	n.a
	Voltinism	n.a	= 0.004	n.a	–	n.a	= 0.022	n.a	= 0.001	n.a	< 0.001
	Sociality	n.a	< 0.001	n.a	= 0.008	n.a	= 0.028	n.a	= 0.004	n.a	< 0.001
	Nesting	n.a	< 0.001	n.a	= 0.033	n.a	–	n.a	= 0.007	n.a	–
	Lecticism	n.a	= 0.016	n.a	–	n.a	–	n.a	= 0.012	n.a	–
	Foraging range	n.a	= 0.021	n.a	–	n.a	–	n.a	–	n.a	–

Legend. n.a.: not available value (not tested variable); -: non-significant p-value.

#### DWV models

The occurrence and load of DWV were significantly negatively related to temperature and relative humidity. The GLM models showed that the probability of DWV occurrence was also significantly related to latitude. In particular, there was a high significant probability of DWV occurrence in South Italy. The probability of DWV occurrence and load was significantly higher in cuckoo bees compared to wild bees and honey bees. There was a significant interaction between temperature and relative humidity in relation to DWV infections. Specifically, high temperatures and lower humidity increased the probability of infections. There was another significant interaction between apiary density and environmental management. In particular, in a semi-natural environment, the probability of infections increased with higher density of the apiaries.

In wild bees, the probability of infection and relative load were significantly related to functional traits: voltinism, sociality, nesting and lectism Specifically, bivoltine bees were significantly more susceptible to infection compared to univoltine bees. Primitively eusocial bees were significantly less likely to be infected than solitary bees and communal bees. Excavator bees were significantly more susceptible to infection compared to carder and renter bees. Oligolectic bees were significantly more likely to be infected than polylectic bees. Also, DWV load in LMER models was significantly negatively related to the foraging range.

#### BQCV models

The occurrence of BQCV was significantly correlated with the apiary density, in particular, the occurrence decreased with the increase in apiary density. Also, the occurrence of BQCV was significantly positively correlated with relative humidity. The probability of infections was significantly lower in the South compared to North or Central Italy, while the infection of BQCV was significantly higher in bees sampled in areas with intensively managed soils than in areas with semi-natural management. The interaction between temperature and relative humidity was significantly correlated with the BQCV occurrence, specifically, there was a high probability of infection with lower temperatures and higher relative humidity. The BQCV load was significantly negatively correlated to temperature. The interaction between temperature and environmental management was significantly correlated with the viral load: the risk to contract a high load of BQCV was higher in low temperatures where bees were sampled in environments with intensive management.

The probability of infection for wild bees was significantly correlated to functional traits: voltinism, sociality, construction of the nest and diet specialization, as well as the BQCV load. The BQCV occurrence and load were significantly higher for bivoltine bees compared to univoltine bees, while the probability of infection was significantly lower for primitively eusocial bees related to solitary bees, especially compared to communal bees. Renter bees were significantly less susceptible to infection than excavator bees, whereas oligolectic bees were significantly more susceptible to infection than polylectic bees, as well as for BQCV load.

#### CBPV models

The occurrence and load of CBPV were significantly negatively correlated with temperature and significantly positively correlated with relative humidity. The probability of infection and abundance were significantly higher for honey bees compared to wild bees.

The probability of CBPV infection in wild bees was significantly correlated with environmental management, specifically, there was a high probability to contract the infection in environments with intensive management. The occurrence of CBPV in wild bees was significantly correlated with functional traits voltinism and sociality. In particular, bivoltine bees were significantly more susceptible to infection compared to univoltine bees, while communal bees were significantly more susceptible to infection compared to primitively eusocial bees, such as for the CBPV load.

#### ABPV models

The occurrence of ABPV was significantly positively correlated with the apiary density. The probability of infection and the load were significantly negatively correlated with temperature and relative humidity. The probability to contract infections of ABPV was significantly lower in Central Italy compared to North and South Italy, while this probability was significantly higher for bees sampled in areas with semi-natural management compared to intensive management. There was a significant interaction between temperature and relative humidity related to ABPV occurrence, in particular, the probability of infection increased at lower temperatures and lower relative humidity, as well as for abundance. The occurrence of ABPV was significantly correlated with the interaction between apiary density and environmental management. In detail, the risk of infection increased with the increase of apiary density in semi-natural environments. Another significant interaction between temperature and environmental management showed the increase of ABPV occurrence and load when temperatures decrease in semi-natural environments. Cuckoo bees were significantly highly susceptible to ABPV infections compared to wild bees and honey bees, such as for ABPV abundance.

The probability of infection was significantly correlated to voltinism and sociality. In particular, bivoltine bees were significantly less susceptible to ABPV infection compared to univoltine bees. Also, primitively eusocial bees were significantly less susceptible to ABPV infection compared to solitary bees, the same was found for abundance. In addition, in the LMER model excavator bees were significantly more likely to have a high viral load of ABPV compared to renter bees.

#### N. ceranae models

The occurrence and load of *N. ceranae* were significantly negatively correlated to temperature and relative humidity. There was a significant interaction between temperature and relative humidity, in particular, the risk of infection decreased much more at high relative humidity when the temperatures increased. In addition, in the LMER model honey bees were significantly more likely to have a high load of *N. ceranae* compared to wild bees.

The probability of infection in wild bees was significantly negatively correlated with apiary density. The risk of infection was significantly correlated with functional traits: voltinism, sociality, construction of the nest and diet specialization. Bivoltine bees were significantly more susceptible to *N. ceranae* compared to univoltine bees, as reported for *N. ceranae* abundance. The probability of *N. ceranae* occurrence and load were significantly higher for solitary bees compared to primitively eusocial bees. The probability of infection was significantly lower for renter bees compared to excavator bees, while this probability was significantly higher for oligolectic compared to polylectic bees.

### Co-infections

**Figure 5:**
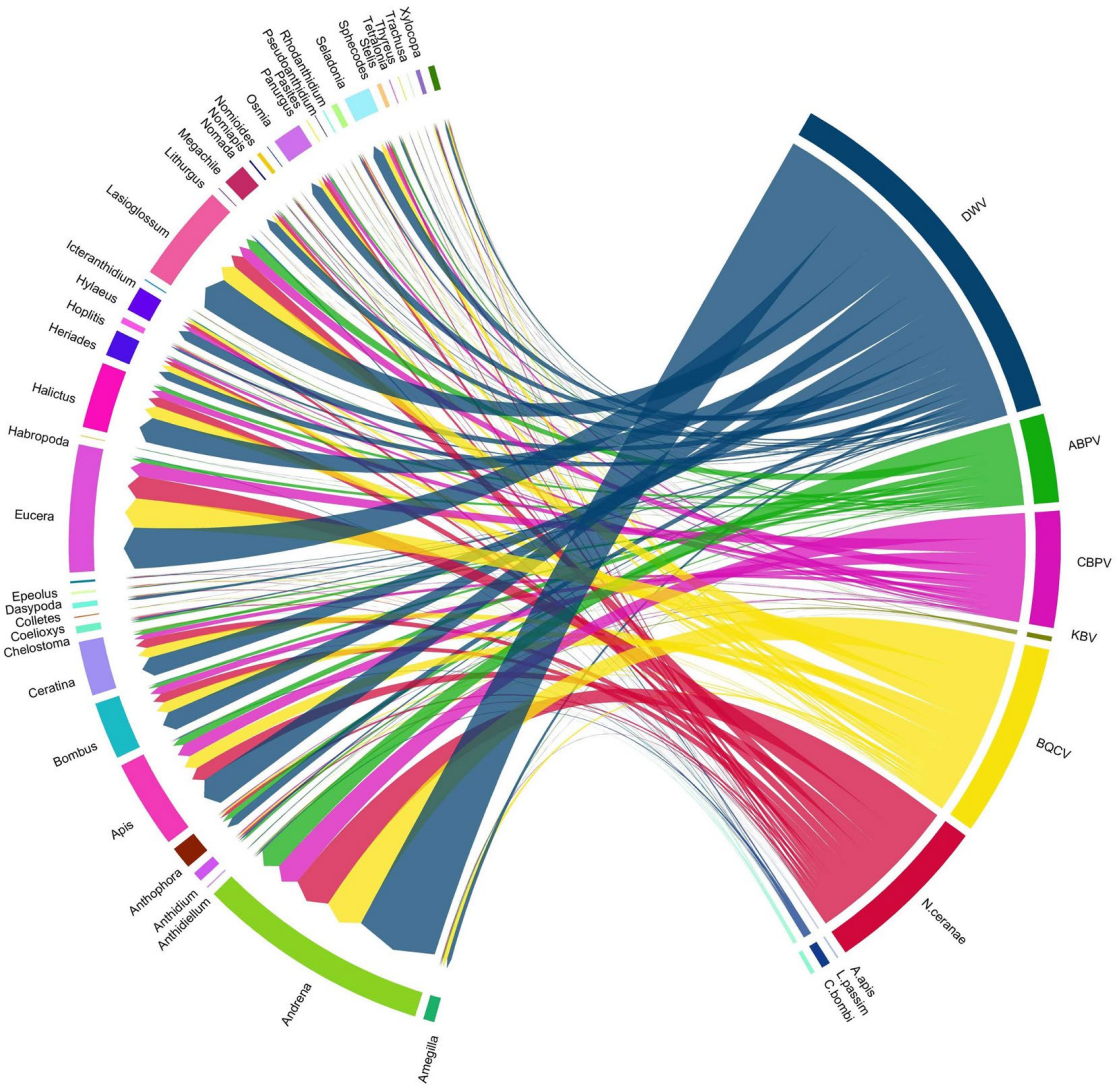
A visual schematization of the investigated pathogens that were involved in multiple infections related to the genera implicated. The arrow thickness denotes the number of co-infections observed within the same host genera. In the graph only bees infected with at least two pathogens appear.

A total of 1365 bees (40.5%) belonging to 173 different species were found to be co-infected with two or more pathogens. Eight infected individuals were detected with five pathogens concurrently. In particular, the co-infection of DWV, BQCV, ABPV, CBPV and *N. ceranae* was detected in two individuals of *Eucera eucnemidea* Dours, 1873, one *Eucera nigrifacies* Lepeletier, 1841, one *Chelostoma florisomne* (L., 1758) and one *Ceratina cucurbitina* (Rossi, 1792). One individual of *Eucera vulpes* (Brullé, 1832) was detected with a co-infection of DWV, BQCV, CPBV, *N. ceranae* and *A. apis*. An individual of *Andrena impunctata* Pérez, 1895 was co-infected with DWV, BQCV, CBPV, *N. ceranae* and *C. bombi*. One individual of *Andrena humilis* Imhoff, 1832 was detected with a co-infection of DWV, BQCV, CBPV, *N. ceranae* and *L. passim*. The genera with the highest number of multiple infections were *Andrena* (Fabricius, 1775), *Eucera* (Scopoli, 1770), *Lasioglossum* (Curtis, 1833), *Apis mellifera* L. and *Halictus* (Latreille, 1804) (Fig. 5).

## Discussion

There is very little information about the detection of pathogens among wild bees in Italy. This is the first study providing large-scale monitoring, involving 11 out of 20 Italian regions. A pilot study was previously conducted in 2021 in the same sites in Emilia-Romagna and Piedmont regions highlighting the infection of 13 pathogens on investigated wild pollinators^21^. In Italy, other studies have confirmed the presence of honey bee viruses in *Vespa velutina* Lepeletier, 1836 and *V. orientalis* L., 1771, probably infected by eating infected honey bees^34,35,76,77^. In addition, a queen of the hornet *V. crabro* L., 1761 was found to be symptomatic for DWV, showing short and crippled wings^76^. The possible effects and symptoms of these pathogens on new hosts are mostly unknown and there are insufficient data to define the real impact of pathogens on wild bee communities^28,78–81^.

In this study, 68.4% of the sampled specimens tested positive for at least one pathogen. This result is in line with previous investigations conducted in Italy, France and U.S.A., with 69.3%, 79% and 80.4% of samples scored infected, respectively^21,78,82,83^.

The highest prevalence was found for DWV, BQCV and *N. ceranae* (51.5%, 26.6% and 22.7% respectively). DWV was the most prevalent pathogen. This virus is widespread in several arthropod species, indicating its strong adaptability to new hosts^18,21,84^ and its generalist nature^24,78,84,85^. BQCV is a less studied but globally distributed virus among honey bees^86^. In 2016 it was found to be highly prevalent (75%) within Italian honey bee colonies^87^, while in Croatian bumblebees a prevalence of 58.5% and 88.9% was detected^19,88^. *N. ceranae* is also a highly adaptive pathogen and it was found in several arthropod species^18,64,89–91^.

The viruses CBPV and ABPV were also found with a prevalence of 16% and 12.1% respectively. Both viruses were detected in other hymenopterans. In France, 66.7% of investigated samples were infected by ABPV, while CBPV was not detected^82^. On the other hand, in Croatia, both viruses were detected in 3.7% of the bumblebees^88^, while in Italy, the prevalence of the two viruses was 9.5% and 5.5%, respectively^21^. This frequency could be linked to the infection of both viruses in the honey bee colonies in which they have specific seasonal trends and epidemiology^87,92–94^.

Currently, information on the presence of KBV, trypanosomatids and *A. apis* in Italy is still scarce. In this study, the presence of *C. mellificae* was not found, while *L. passim, C. bombi,* KBV and *A. apis* were also found with a prevalence of 1.2%, 0.6%, 0.7% and 0.2%, respectively. In Italy, the presence of *C. mellificae* had been detected for the first time in 2014 in one apiary in the Lazio region^95^, followed by another identification in the Veneto region in 2020^96^. However, in the Veneto region, the presence of *L. passim* was found to be 48.8% and 62.2% in 2020 and 2021, respectively^96^, while no presence was detected in the Emilia-Romagna region^97^. The first Italian identification of KBV was notified in the Lazio region^98^, and only a few cases were detected in Italy^87^. Among pollinators, KBV and *A. apis* prevalence were respectively 0.7% and 0.2%, finding positive bees and syrphids^21^, in line with the results of this study.

Most pathogens averaged an abundance per individual of less than 10 × 10^5^, which is lower than the threshold generally linked to symptomatic infection in *A. mellifera* (10 × 10^6^)^21,28,99–101^. DWV was the virus with the highest mean abundance, with an average viral load of 10 × 10^10^. Besides, a high average abundance was found for BQCV, with an average viral load of 10 × 10^9^. For CBPV the average viral load was found to be 10 × 10^6^. These findings indicated a widespread infection among wild bees that may be related to the high prevalence of all three viruses among honey bee colonies and their spread in the environment^80,87,92–94,102–105^.

The trends of the five most prevalent pathogens (DWV, BQCV, CBPV, ABPV and *N. ceranae*) were in line with the same seasonal trends in honey bee colonies^21,87,99,106^. In honey bees, these pathogens show an initial increase in cases of infection during early spring with a peak in late spring/early summer when the number of workers is very high, and in many cases triggered by *Varroa* infestation^21,87,107–109^. In this study, it is interesting to note the decrease in cases of infection in the middle of the spring period; this could be related to a possible dilution phenomenon, where an increase in the biodiversity of pollinator species could promote a "thinning" of pathogens among bees^43,110^. In addition, greater biodiversity and availability in terms of flower species could reduce pathogen transmission among bees^30,43,111^. The peak of pathogen abundance reached in June may be related both to population dynamics within the honey bee colony, where in this month the colony reaches maximum density and activity, but also to the availability of floral resources^87,99,106,111^.

The inclusion of meteorological, environmental, and biological factors in the statistical models could be considered as successful predictors to the infection risk among bees. The increase in temperature and relative humidity suggests the risk of infection reduction, probably related to the host ecology and the persistence of the pathogen in the environment^20^. In fact, UV levels can inactivate viral particles in flowers, and the increase in temperature together with humidity can reduce the permanence of pathogens in the environment^20,40,41,112,113^. In addition, the high temperatures reduced the flowers anthesis causing a decrease in bees foraging activity^114,115^, causing also oxidative stress and mortality^116,117^.

In this study, apiary density was related to an increasing ABPV infection risk, probably linked to the in-hive spread of the virus by *Varroa* bite^23,118,119^. On the other hand, apiary density has a negative effect on the risk of BQCV and *N. ceranae* infection. BQCV is mainly related to honey bee colonies affecting queen brood cells and it can be assumed that it is related to eusociality, as reported also for bumblebees^19,88,105,120,121^. Besides, *N. ceranae* spores can remain quiescent in the environment for many years, replicating in the host after ingestion^87,122,123^.

The probability of infection depended on latitude, probably related to the different meteorological conditions present in Italy. Southern Italy is characterized by a Mediterranean climate, while northern and central Italy ranges from a humid subtropical to a humid continental climate^124^. Different infections related to latitude may be linked to the presence of different pathogens’ genetic variants and their environmental-adapted circulation in specific geographical areas^18,21,103^.

The types of agricultural environmental management have a different association with the risk of infection, depending on the pathogen. The increase of BQCV and CPBV infection in intensively managed environments could be linked to the synchronized bloom events of monocultures, leading to a high density of bees in a specific area and consequently exacerbating the spread of pathogens^37,39,43^. Instead, the risk of ABPV infection in a semi-natural environment could be related to the presence of honey bees, because the massive use of pesticides in intensive agricultural areas could discourage beekeeping activity in them^23,37,119^.

The biological and social traits of bees were found to play a key role in the risk of infection. DWV and ABPV risks were higher in cuckoo bees. Their cleptoparasitic behavior toward their hosts, such as robbing of food resources and exploitation of parental care, may exacerbate the horizontal transmission^23,24^. Although in other studies sociality was not found to be relevant in the transmission of pathogens^82,125^, in this work, primitively eusocial bees presented a lower risk of infection compared to communal bees. In communal bees, multiple reproductive females share the same nest and lay their eggs, this aggregation could promote the horizontal and passive spread of viruses^37,126^. However, in primitively eusocial bees, specific behavioural or physiological immunity could help to maintain the health of the nest^127–129^. For example, newly-emerged *B. terrestris* (L. 1758) individuals may improve their resistance against *C. bombi* by feeding with faeces to obtain bacterial microbiota^130^, even if there are still little information available for social species, excluding honey bees.

Bivoltine bees and solitary excavator bees were found to be more susceptible to pathogen infection, most likely due to their reproductive cycle and nest location. The generation of two broods per year could intensify vertical transmission^37,131^. Often in such cases, the same nests are used for the next brood, facilitating the pathogen transmission between generations^132^, even if the influence of nest characteristics on susceptibility to pathogens remains unclear. However, less sun exposure on the ground and low temperatures may favour the persistence of viral particles in the soil and the resistance of *N. ceranae* spores^42,123,132^. In addition, many pesticides used in agriculture remain bound to soil particles from a few months to many years and several studies have shown that these products can affect the immunocompetence of bees^132,133^.

In this study, a significant correlation was found between *N. ceranae* and BQCV. Both pathogens could interact together promoting co-infections, and causing a synergistic effect on honey bee mortality^134–137^. The association between DWV and *N. ceranae* could also cause a synergistic effect, accelerating the DWV replication in honey bees^138^. A significant negative correlation was observed between ABPV and BQCV, hypnotizing a possible competition between these viruses, able to co-infect bees^139^. The significant and positive correlation between DWV and BQCV confirms the possible interaction of both viruses in the hosts, even if the synergistic effects remain still unclear^140^.

In this study, a high number of co-infected individuals were detected. There are several researches on the prevalence of pathogens in bees that report multiple infections, and it is often common in the natural environment^18,21,102,137,141–144^.

## Conclusion

This study extended our knowledge of the epidemiological situation of honey bee pathogens in 11 Italian regions and their circulation among wild bees. The use of models has been fundamental in predicting the risk of infection among bees and in understanding which social and ecological factors influence pathogen interspecific occurrence. Although the investigated pathogens are known as specific to the honey bee for historical reasons, and the most prevalent hypothesis is that this species may act as a reservoir, the directionality of the transfer is difficult to identify and prove. It appears that only the spread of some pathogens is correlated with the presence of the honey bees, while for other pathogens there is already active circulation between different bee species. The share of the same environment and food resources could increase the pathogens' transmission, although further studies are needed to clarify these dynamics.

The most emerging aspect is that some bee species may be more easily susceptible than others, due to their biological and behavioural features. However, the high lack of data for many species makes it difficult to assess the impact of pathogens on these populations. Besides, the effects and symptoms of pathogens infection in wild bees are still lacking, and further studies on fitness, behaviour and development are needed to increase conservation efforts of wild bees.

This study provided evidence of a strong relationship between the health of the environment, pollinators and human activities, as part of a One Health approach, which is essential to protect the features and functionality of ecosystems.

### Supplementary Information

Supplementary Table S1.

Supplementary Table S2.

Supplementary Table S3.

Supplementary Table S4.

Supplementary Table S5.

Supplementary Table S6.

Supplementary Table S7.

Supplementary Table S8.

Supplementary Table S9.

Supplementary Figure S1.

Supplementary Figure S2.

Supplementary Figure S3.

Supplementary Figure S4.

Supplementary Figure S5.

## Data Availability

All data generated and analysed in this study are included in this published article and in its related supplementary information files.
