# Supplementary material for: Ecological and social factors influence interspecific pathogens occurrence among bees
Source: Sci Rep. 2024 Mar 1;14:5136. doi: 10.1038/s41598-024-55718-x (PMC10907577; doi:10.1038/s41598-024-55718-x)
Supplement: Supplementary file 1 — Supplementary Table S1. [file 41598_2024_55718_MOESM1_ESM.docx]

*Table S1. Geographical and environmental characterization of sampling sites.*

| Site acronym | Agro-ecosystem | Region | City/Town | Province |
| --- | --- | --- | --- | --- |
| ABAI | Intensive | Abruzzo | Fonte Ventola | Pescara |
| ABES | Semi-natural | Abruzzo | Colle Bianco | Pescara |
| CAAI | Intensive | Campania | Paestum | Salerno |
| CAES | Semi-natural | Campania | Caselle in Pittari | Salerno |
| ERAI | Intensive | Emilia-Romagna | Massa Castello | Ravenna |
| ERES | Semi-natural | Emilia-Romagna | Rocca San Casciano | Forlì-Cesena |
| ERESP | Semi-natural | Emilia-Romagna | San Lazzaro | Bologna |
| FRAI | Intensive | Friuli Venezia Giulia | Udine | Udine |
| FRES | Semi-natural | Friuli Venezia Giulia | Forgaria nel Friuli | Udine |
| PIAI | Intensive | Piedmont | Cherasco | Cuneo |
| PIES | Semi-natural | Piedmont | Baldissero d’Alba | Cuneo |
| PUAI | Intensive | Apulia | Polignano a Mare | Bari |
| PUES | Semi-natural | Apulia | Ruvo di Puglia | Bari |
| SAAI | Intensive | Sardinia | Ottava | Sassari |
| SAES | Semi-natural | Sardinia | Porto Ferro | Sassari |
| SIAI | Intensive | Sicily | Spinasanta | Catania |
| SIES | Semi-natural | Sicily | Torre Allegra | Catania |
| TOAIPI | Intensive | Tuscany | San Rossore | Pisa |
| TOAIRO | Intensive | Tuscany | Rosignano | Livorno |
| TOESRO | Semi-natural | Tuscany | Rosignano | Livorno |
| UMAI | Intensive | Umbria | Mercatello | Perugia |
| UMES | Semi-natural | Umbria | Assisi | Perugia |
| VEAI | Intensive | Veneto | Pojana Maggiore | Vicenza |
| VEES | Semi-natural | Veneto | Teolo | Padova |
