# Supplementary material for: Ecological and social factors influence interspecific pathogens occurrence among bees
Source: Sci Rep. 2024 Mar 1;14:5136. doi: 10.1038/s41598-024-55718-x (PMC10907577; doi:10.1038/s41598-024-55718-x)
Supplement: Supplementary file 2 — Supplementary Table S2. [file 41598_2024_55718_MOESM2_ESM.docx]

*Table S2. List of primers used to detect fungus, microsporidian, trypanosomatids and viruses.*

| Target | Primer name | Sequence (5’-3’) | Temperature of annealing | Reference |
| --- | --- | --- | --- | --- |
| *Ascosphaera apis* | A_apis_3-F1  A_apis_3-R1 | TGTCTGTGCGGCTAGGTG  CCACTAGAAGTAAATGATGGTTAGA | 62°C | (James and Skinner, 2005) |
| *Nosema ceranae* | Hsp70_F  Hsp70_R | GGGATTACAAGTGCTTAGAGTGATT  TGTCAAGCCCATAAGCAAGTG | 63°C | (Cilia et al., 2018a) |
| *Lotmaria passim* | Lp2F 459  Lp2R 459 | AGGGATATTTAAACCCATCGAA  ACCACAAGAGTACGGAATGC | 60°C | (Arismendi et al., 2016) |
| *Crithidia mellificae* | Cmel_Cyt_b_F  Cmel_Cyt_b_R | TAAATTCACTACCTCAAATTCAATAACATAATCAT  ATTTATTGTTGTAATCGGTTTTATTGGATATGT | 60°C | (Xu et al., 2018) |
| *Crithidia bombi* | C.bombi_119Fw  C.bombi_119Rv | CCAACGGTGAGCCGCATTCAGT  CGCGTGTCGCCCAGAACATTGA | 64.5°C | (Huang et al., 2015) |
| DWV | DWV Fw 8450  DWV Rev 8953 | TGGCATGCCTTGTTCACCGT  CGTGCAGCTCGATAGGATGCCA | 60°C | (Mazzei et al., 2018) |
| BQCV | BQCV 9195F  BQCV 8265R | GGTGCGGGAGATGATATGGA  GCCGTCTGAGATGCATGAATAC | 60°C | (Chantawannakul et al., 2006) |
| CBPV | CPV 304F 79  CPV 371R | TCTGGCTCTGTCTTCGCAAA  GATACCGTCGTCACCCTCATG | 60°C | (Chantawannakul et al., 2006) |
| ABPV | APV 95F  APV 159R | TCCTATATCGACGACGAAAGACAA  GCGCTTTAATTCCATCCAATTGA | 60°C | (Chantawannakul et al., 2006) |
| KBV | KBV 83F  KBV 161R | ACCAGGAAGTATTCCCATGGTAAG  TGGAGCTATGGTTCCGTTCAG | 60°C | (Chantawannakul et al., 2006) |

DWV, deformed wing virus; BQCV, black queen cell virus; CBPV, chronic bee paralysis virus; ABPV, acute bee paralysis virus; KBV, Kashmir bee virus.
