# Supplementary material for: Ecological and social factors influence interspecific pathogens occurrence among bees
Source: Sci Rep. 2024 Mar 1;14:5136. doi: 10.1038/s41598-024-55718-x (PMC10907577; doi:10.1038/s41598-024-55718-x)
Supplement: Supplementary file 4 — Supplementary Table S4. [file 41598_2024_55718_MOESM4_ESM.docx]

| **Region** | **Sampling Site** | **Month** | | | | | | | | **Total per transect** | **Total per region** |
| --- | --- | --- | --- | --- | --- | --- | --- | --- | --- | --- | --- |
|  |  | **February** | **March** | **April** | **May** | **June** | **July** | **August** | **September** |  |  |
| **Abruzzo** | ABAI |  | 9 |  | 15 |  | 23 |  | 10 | **57** | **102** |
|  | ABES |  |  |  | 18 |  | 14 |  | 13 | **45** |  |
| **Campania** | CAAI |  |  | 68 | 41 | 28 | 37 | 47 |  | **221** | **430** |
|  | CAES |  |  | 39 | 36 | 35 | 45 | 38 | 16 | **209** |  |
| **Emilia-Romagna** | ERAI |  | 8 | 35 | 31 | 50 | 30 | 15 | 19 | **188** | **508** |
|  | ERES |  |  | 65 | 68 | 12 | 24 | 22 | 14 | **205** |  |
|  | ERESP |  | 15 | 27 | 11 | 19 | 28 | 15 |  | **115** |  |
| **Friuli-Venezia Giulia** | FRAI |  |  | 5 | 10 | 22 | 26 | 37 | 14 | **114** | **272** |
|  | FRES |  |  | 4 | 18 | 31 | 51 | 30 | 24 | **158** |  |
| **Piedmont** | PIAI |  |  | 22 | 24 | 10 | 31 | 8 | 12 | **107** | **361** |
|  | PIES |  | 40 | 54 | 31 | 34 | 43 | 37 | 15 | **254** |  |
| **Apulia** | PUAI | 13 |  | 12 | 2 | 5 | 37 | 25 | 16 | **110** | **229** |
|  | PUES | 13 |  |  | 21 |  | 23 | 46 | 16 | **119** |  |
| **Sardinia** | SAAI |  | 21 | 9 | 24 | 17 | 9 | 11 |  | **91** | **160** |
|  | SAES |  | 11 | 13 | 14 | 31 |  |  |  | **69** |  |
| **Sicily** | SIAI | 9 | 23 | 39 | 28 | 9 | 17 | 24 | 25 | **174** | **383** |
|  | SIES | 10 | 18 | 24 | 45 | 37 | 36 | 19 | 20 | **209** |  |
| **Tuscany** | TOAIPI |  | 12 | 44 | 47 | 41 | 24 | 51 | 42 | **261** | **637** |
|  | TOAIRO |  | 30 | 27 | 27 | 29 | 50 | 18 | 25 | **206** |  |
|  | TOESRO |  | 5 | 19 | 21 | 31 | 46 | 27 | 21 | **170** |  |
| **Umbria** | UMAI |  |  |  | 7 | 29 | 11 | 32 | 18 | **97** | **171** |
|  | UMES |  |  | 3 | 11 | 19 | 12 | 14 | 15 | **74** |  |
| **Veneto** | VEAI |  |  | 8 | 14 | 5 | 6 | 16 | 14 | **63** | **119** |
|  | VEES |  |  | 5 | 7 | 4 | 8 | 23 | 9 | **56** |  |
| **Total per month** |  | **45** | **192** | **522** | **192** | **498** | **631** | **555** | **358** | **3372** | |

**Table S4.** The number of samples collected per region, sampling site, and month.
