# Supplementary material for: Ecological and social factors influence interspecific pathogens occurrence among bees
Source: Sci Rep. 2024 Mar 1;14:5136. doi: 10.1038/s41598-024-55718-x (PMC10907577; doi:10.1038/s41598-024-55718-x)
Supplement: Supplementary file 6 — Supplementary Table S6. [file 41598_2024_55718_MOESM6_ESM.docx]

**Table S6.** Detailed post-hoc comparisons of seasonal trends between months per pathogen prevalence. Only significant p-value are shown.

| **Months** | | **DWV p-value** | | **BQCV p-value** | | **CBPV p-value** | | **ABPV p-value** | | ***N. ceranae* p-value** | | ***C. bombi* p-value** | | ***L. passim* p-value** | |
| --- | --- | --- | --- | --- | --- | --- | --- | --- | --- | --- | --- | --- | --- | --- | --- |
| **April** | **August** | < 0.0001 | **** | < 0.0001 | **** | < 0.0001 | **** | < 0.0001 | **** | < 0.0001 | **** |  |  | 0.038 | * |
| **April** | **July** | < 0.0001 | **** | < 0.0001 | **** | < 0.0001 | **** | < 0.0001 | **** | < 0.0001 | **** |  |  | 0.045 | * |
| **April** | **June** |  |  | < 0.0001 | **** | < 0.0001 | **** | < 0.0001 | **** | < 0.0001 | **** |  |  |  |  |
| **April** | **March** |  |  |  |  |  |  |  |  | < 0.0001 | **** |  |  |  |  |
| **April** | **May** |  |  | 0.014 | * |  |  | < 0.0001 | **** |  |  | 0.022 | * |  |  |
| **April** | **September** | < 0.0001 | **** | < 0.001 | **** | < 0.0001 | **** | < 0.0001 | **** | < 0.0001 | **** |  |  | 0.010 | * |
| **August** | **June** | < 0.0001 | **** | < 0.0001 | **** | < 0.0001 | **** |  |  | < 0.0001 | **** |  |  |  |  |
| **August** | **March** | < 0.0001 | **** | < 0.0001 | **** | < 0.0001 | **** | < 0.0001 | **** | < 0.0001 | **** |  |  |  |  |
| **August** | **May** | < 0.0001 | **** | < 0.0001 | **** | < 0.0001 | **** | < 0.0001 | *** | < 0.0001 | **** | 0.004 | ** |  |  |
| **July** | **June** | < 0.0001 | **** | < 0.0001 | **** | < 0.0001 | **** |  |  | < 0.0001 | **** |  |  |  |  |
| **July** | **March** | < 0.0001 | **** | < 0.0001 | **** | < 0.0001 | **** | < 0.0001 | **** | < 0.0001 | **** |  |  |  |  |
| **July** | **May** | < 0.0001 | **** | < 0.0001 | **** | < 0.0001 | **** |  |  | < 0.0001 | **** |  |  |  |  |
| **June** | **March** |  |  |  |  |  |  | < 0.0001 | **** |  |  |  |  |  |  |
| **June** | **May** | 0.014 | ** | < 0.001 | *** | < 0.0001 | **** |  |  | < 0.0001 | **** |  |  |  |  |
| **June** | **September** | < 0.0001 | **** | < 0.0001 | **** | < 0.0001 | **** |  |  | < 0.0001 | **** |  |  |  |  |
| **March** | **May** | 0.016 | ** |  |  |  |  | 0.001 | ** | < 0.0001 | **** |  |  |  |  |
| **March** | **September** | < 0.0001 | **** | < 0.0001 | **** | < 0.0001 | **** | < 0.0001 | **** | < 0.0001 | **** |  |  |  |  |
| **May** | **September** | < 0.0001 | **** | < 0.0001 | **** | < 0.0001 | **** |  |  | < 0.0001 | **** |  |  |  |  |

DWV, deformed wing virus; BQCV, black queen cell virus; CBPV, chronic bee paralysis virus; ABPV, acute bee paralysis virus. Different numbers of asterisks indicate statistical differences from the base average: p-value<0.05 (*); p-value<0.01 (**); p-value<0.001 (***); p-value <0.0001(****).
