# Supplementary material for: Ecological and social factors influence interspecific pathogens occurrence among bees
Source: Sci Rep. 2024 Mar 1;14:5136. doi: 10.1038/s41598-024-55718-x (PMC10907577; doi:10.1038/s41598-024-55718-x)
Supplement: Supplementary file 7 — Supplementary Table S7. [file 41598_2024_55718_MOESM7_ESM.docx]

**Table S7.** The detailed p-value of correlation between pathogens.

|  | **DWV** | **ABPV** | **CBPV** | **KBV** | **BQCV** | ***N. ceranae*** | ***A. apis*** | ***L. passim*** | ***C. bombi*** |
| --- | --- | --- | --- | --- | --- | --- | --- | --- | --- |
| **DWV** | **-** |  |  |  |  |  |  |  |  |
| **ABPV** | <0.001 | **-** |  |  |  |  |  |  |  |
| **CBPV** | <0.001 | 0.03 | **-** |  |  |  |  |  |  |
| **KBV** | 0.561 | 0.971 | 0.366 | **-** |  |  |  |  |  |
| **BQCV** | <0.001 | <0.001 | <0.001 | 0.197 | **-** |  |  |  |  |
| ***N. ceranae*** | <0.001 | 0.855 | <0.001 | 0.710 | <0.001 | - |  |  |  |
| ***A. apis*** | 0.364 | 0.364 | <0.001 | 0.832 | 0.223 | 0.089 | - |  |  |
| ***L. passim*** | 0.165 | 0.527 | 0.949 | 0.578 | 0.258 | 0.373 | 0.786 | - |  |
| ***C. bombi*** | 0.407 | 0.065 | 0.957 | <0.001 | 0.865 | 0.871 | 0.850 | 0.619 | - |

DWV, deformed wing virus; ABPV, acute bee paralysis virus; CBPV, chronic bee paralysis virus; KBV, Kashmir bee virus; BQCV, black queen cell virus.
