# Supplementary material for: Ecological and social factors influence interspecific pathogens occurrence among bees
Source: Sci Rep. 2024 Mar 1;14:5136. doi: 10.1038/s41598-024-55718-x (PMC10907577; doi:10.1038/s41598-024-55718-x)
Supplement: Supplementary file 10 — Supplementary Figure S1. [file 41598_2024_55718_MOESM10_ESM.docx]

| a)  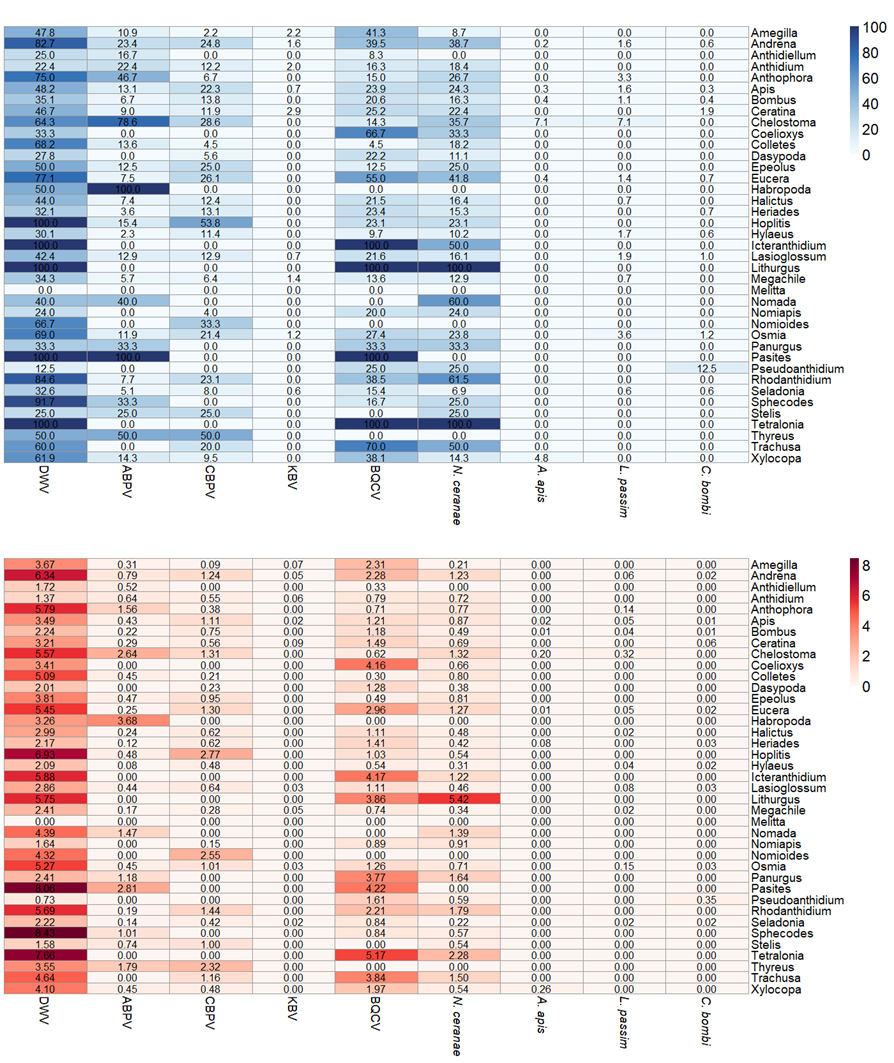 | b)  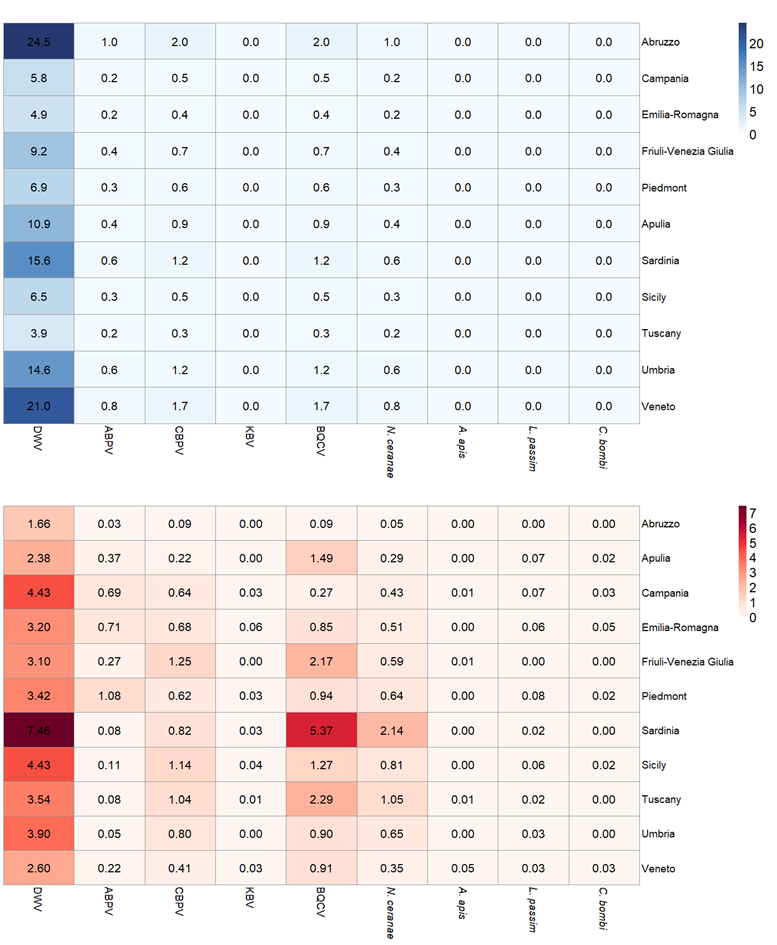 |
| --- | --- |
| c)  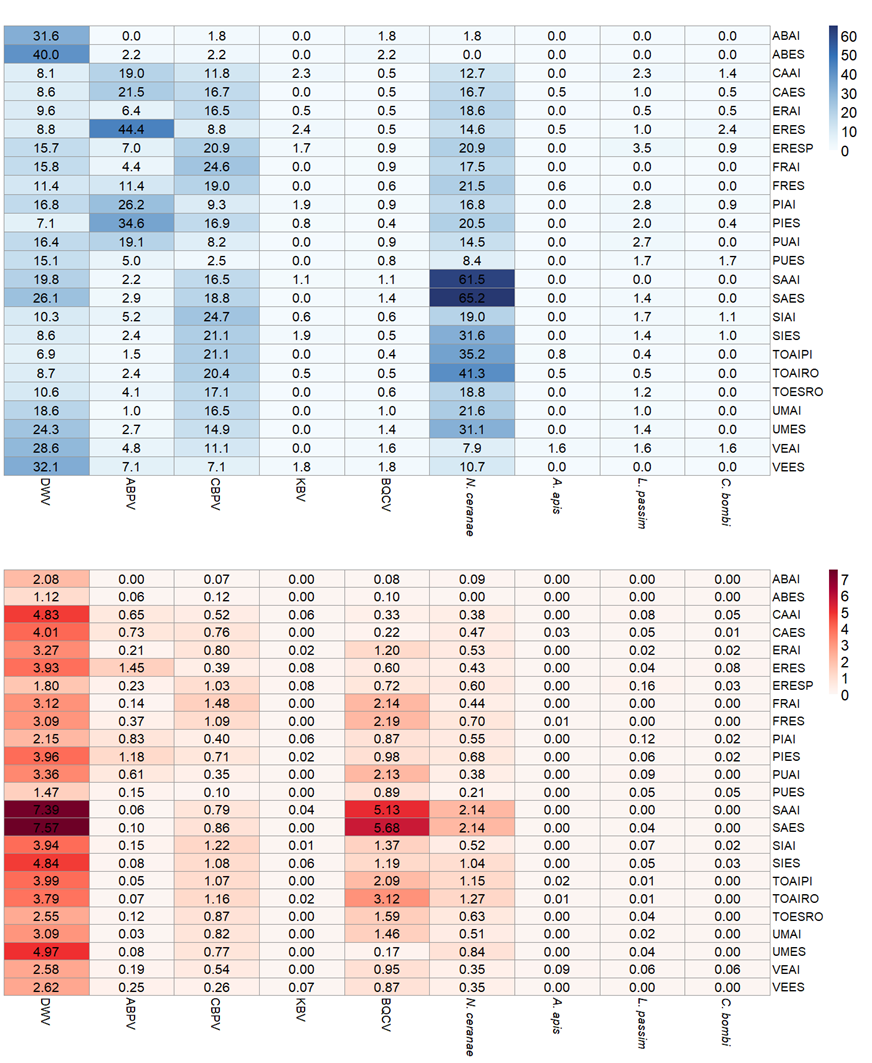 | d)  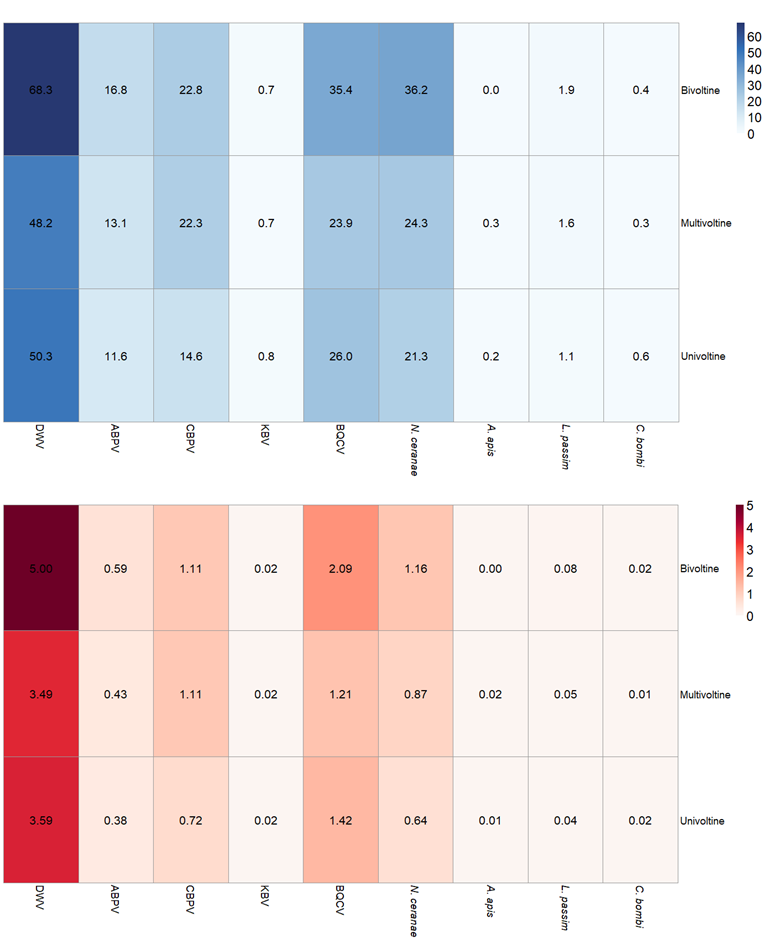 |
| e)  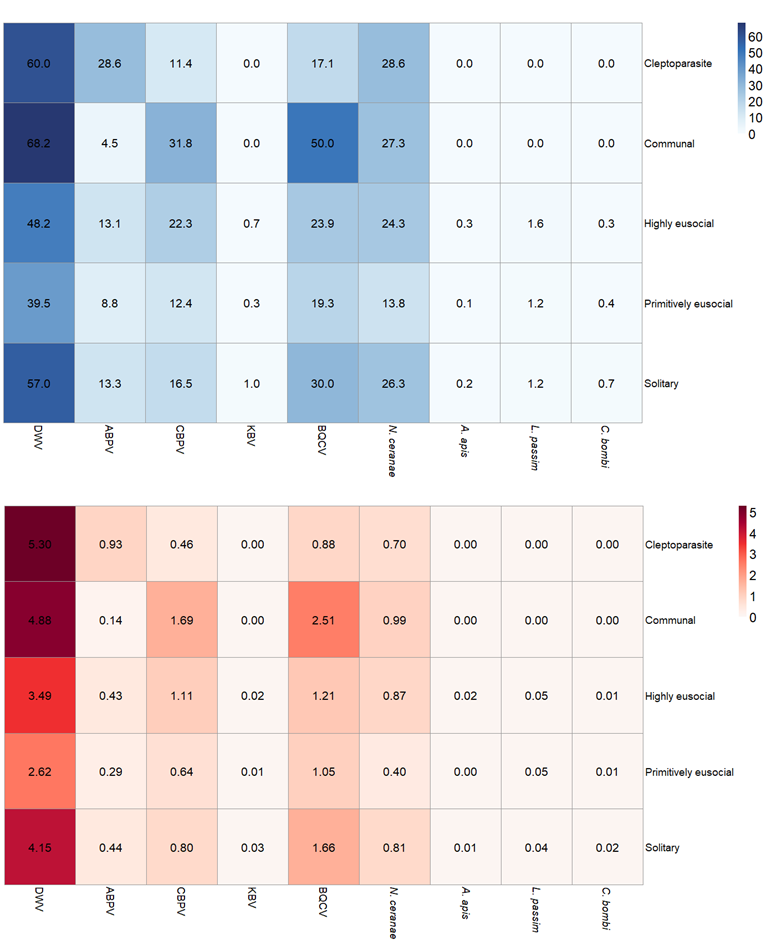 | f)  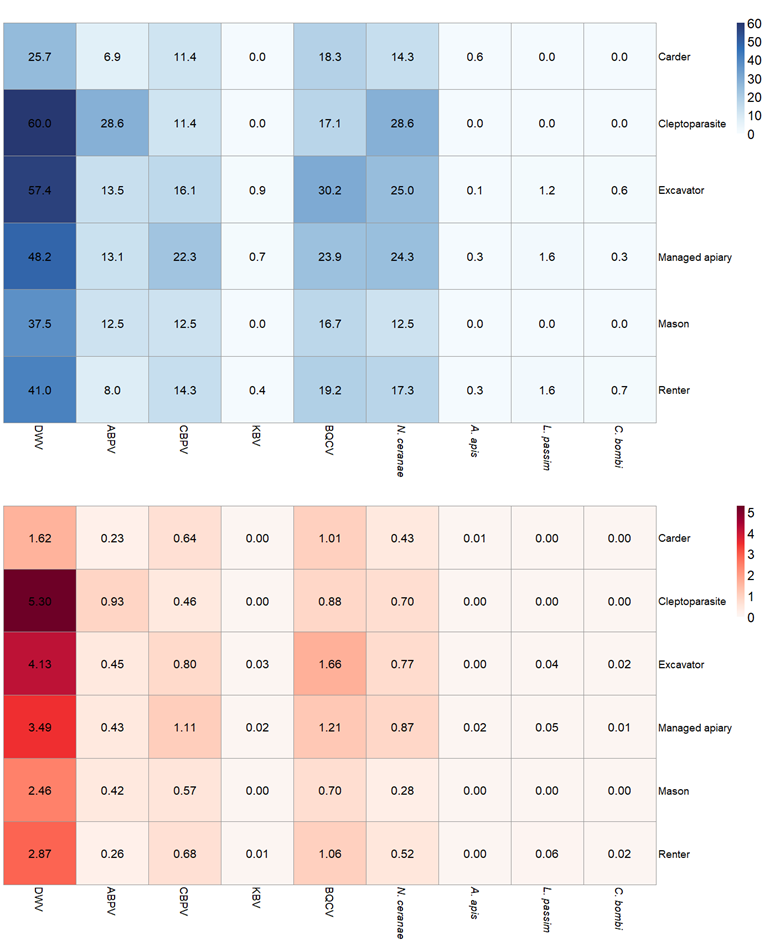 |
| g)  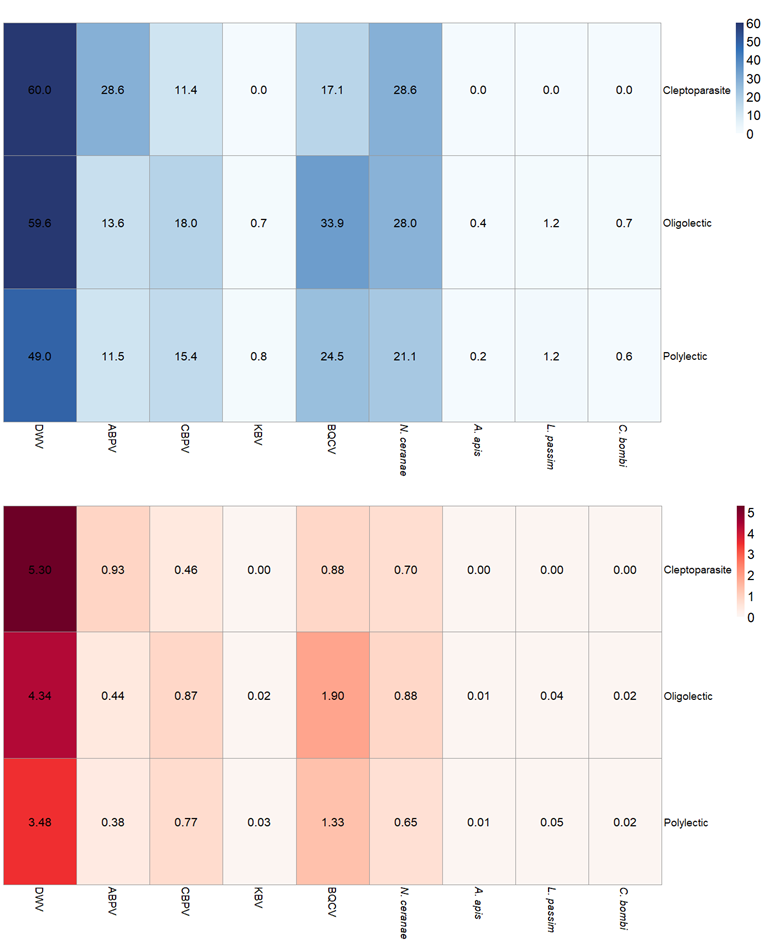 | h)  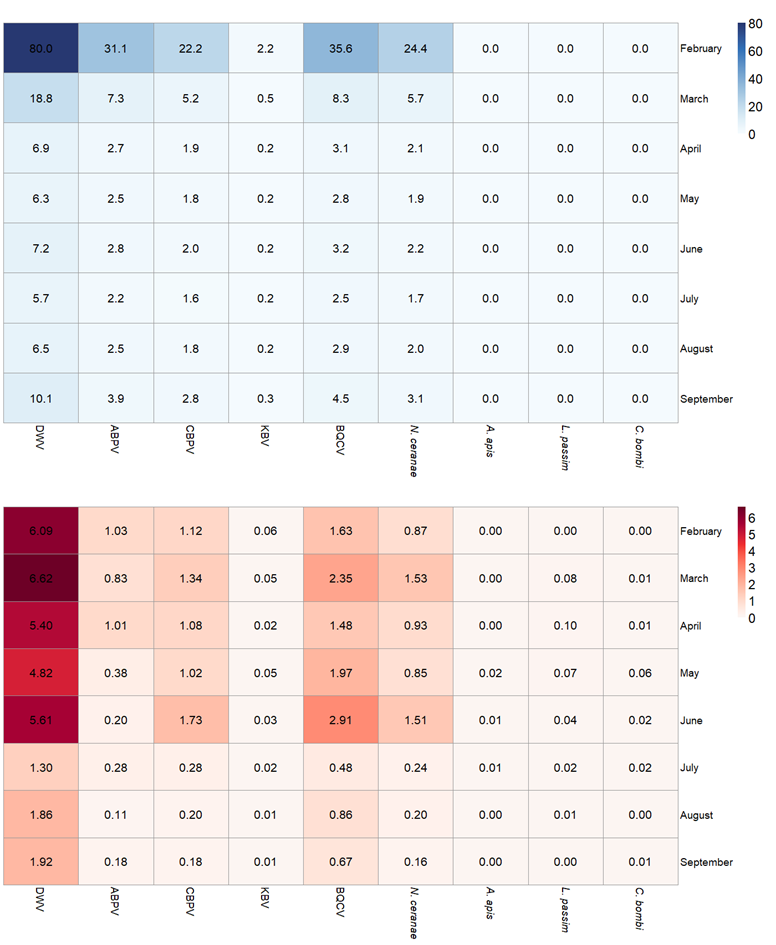 |

Figure S1. a) Prevalence and abundance per genus for the investigated pathogens; b) Prevalence and abundance per region for the investigated pathogens; c) Prevalence and abundance per transect for the investigated pathogens; d) Prevalence and abundance per voltinism for the investigated pathogens; e) Prevalence and abundance per sociality for the investigated pathogens; f) Prevalence and abundance per construction of the nest for the investigated pathogens; g) Prevalence and abundance per diet specialization for the investigated pathogens; h) Prevalence (top graph) and abundance (bottom graph) per month for the investigated pathogens. Prevalence (in blue) is shown as a percentage, while abundance (in red) is shown as a decimal logarithm.
