# Supplementary material for: Ecological and social factors influence interspecific pathogens occurrence among bees
Source: Sci Rep. 2024 Mar 1;14:5136. doi: 10.1038/s41598-024-55718-x (PMC10907577; doi:10.1038/s41598-024-55718-x)
Supplement: Supplementary file 11 — Supplementary Figure S2. [file 41598_2024_55718_MOESM11_ESM.docx]

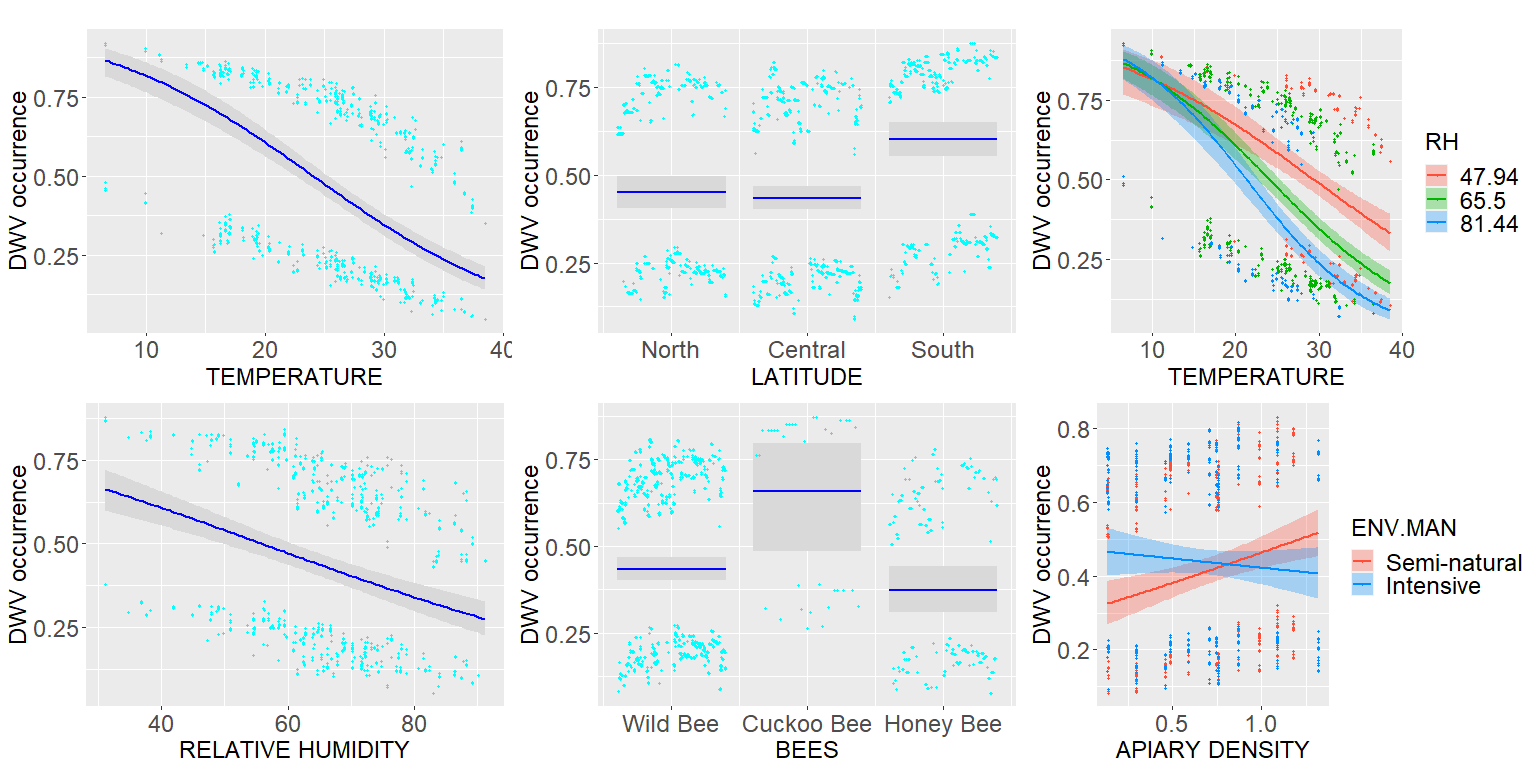


b)

a)


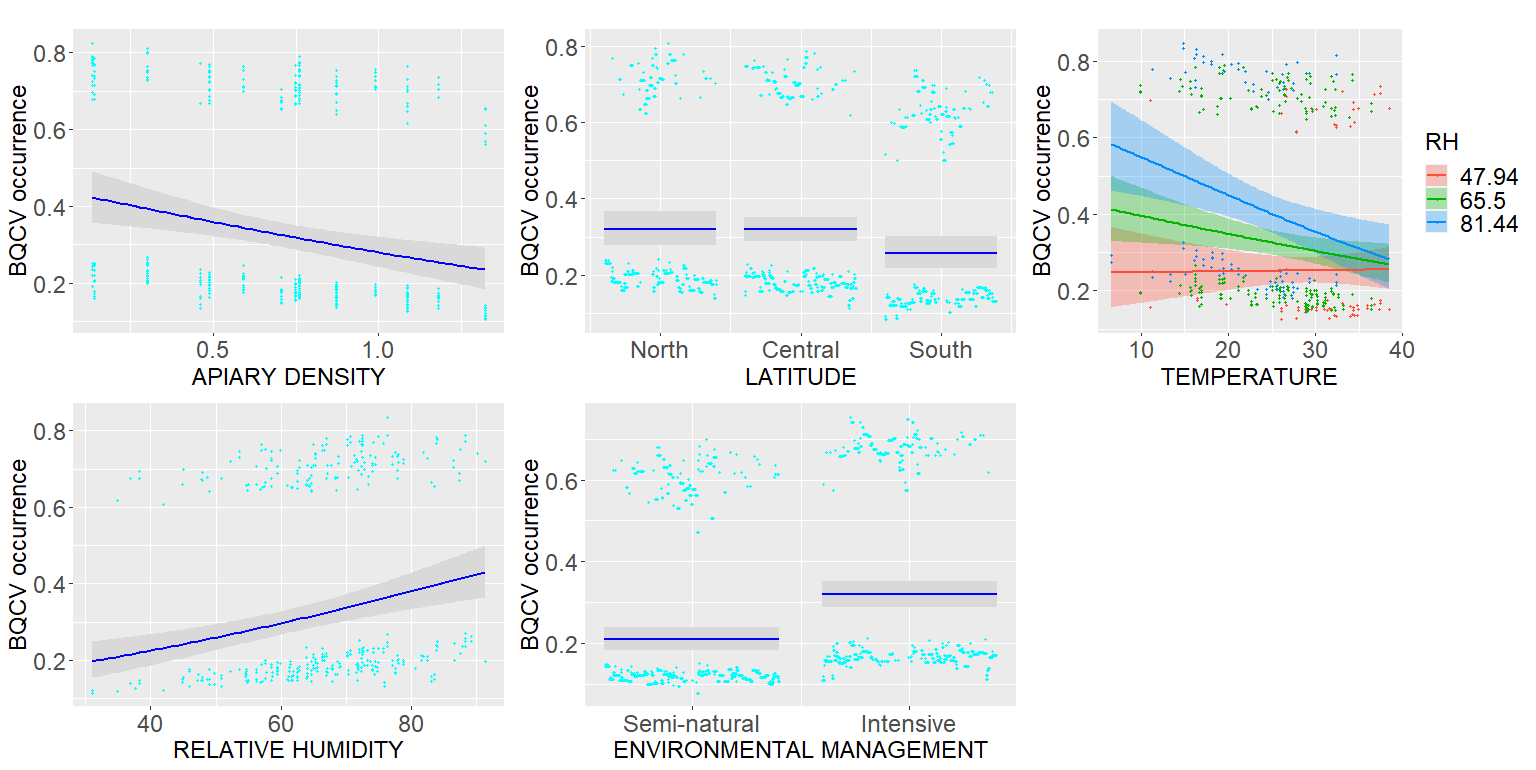


c)


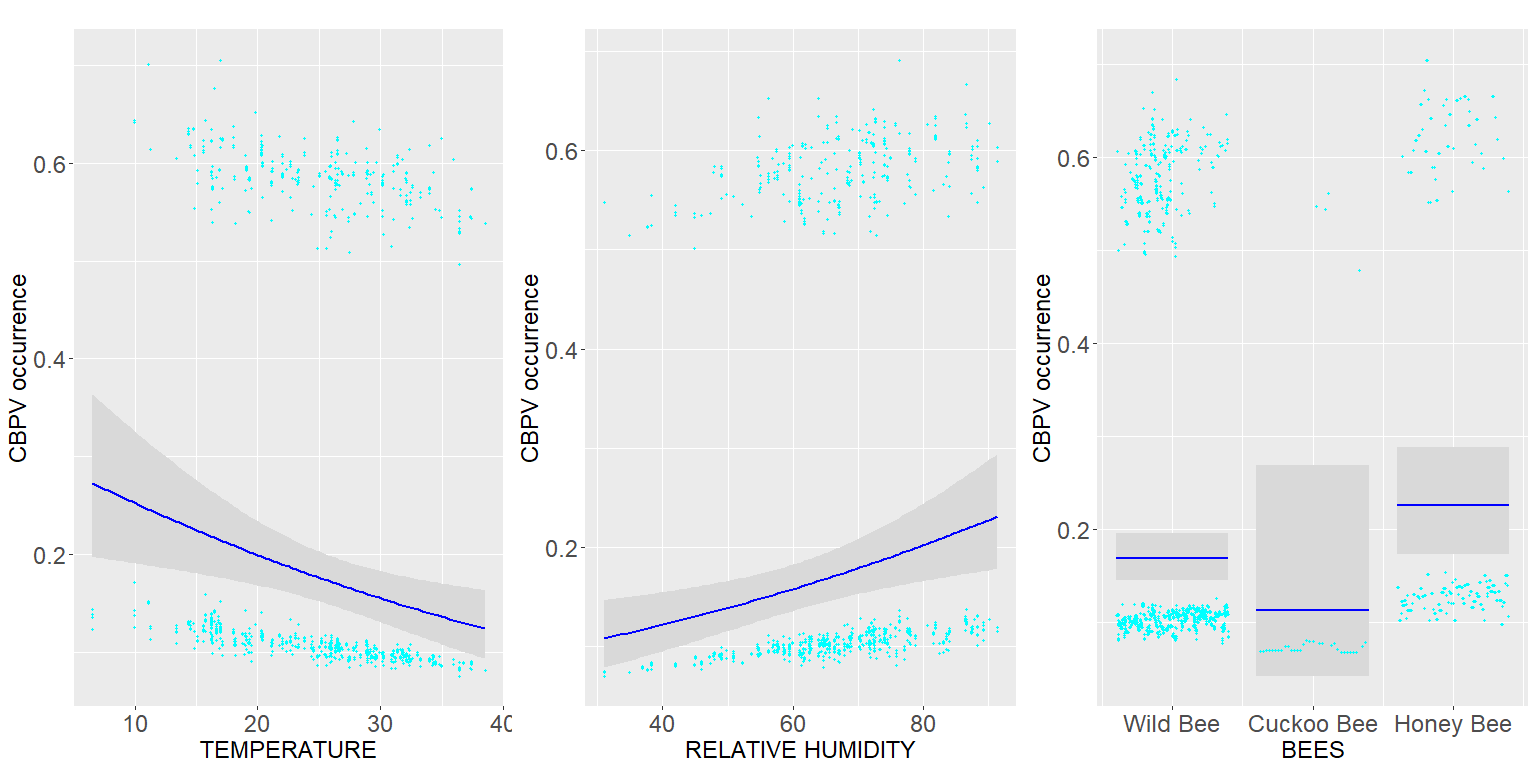


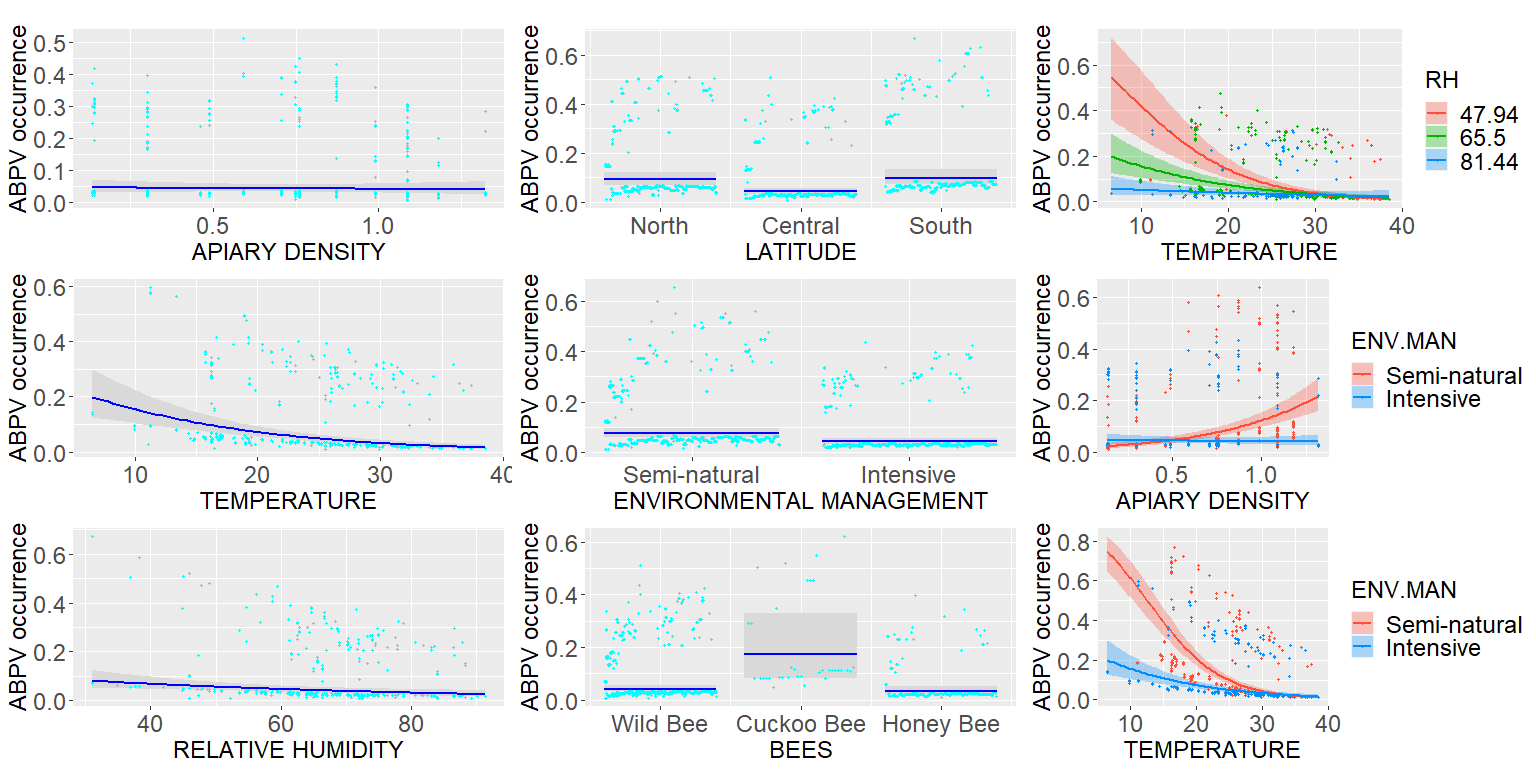


e)

d)


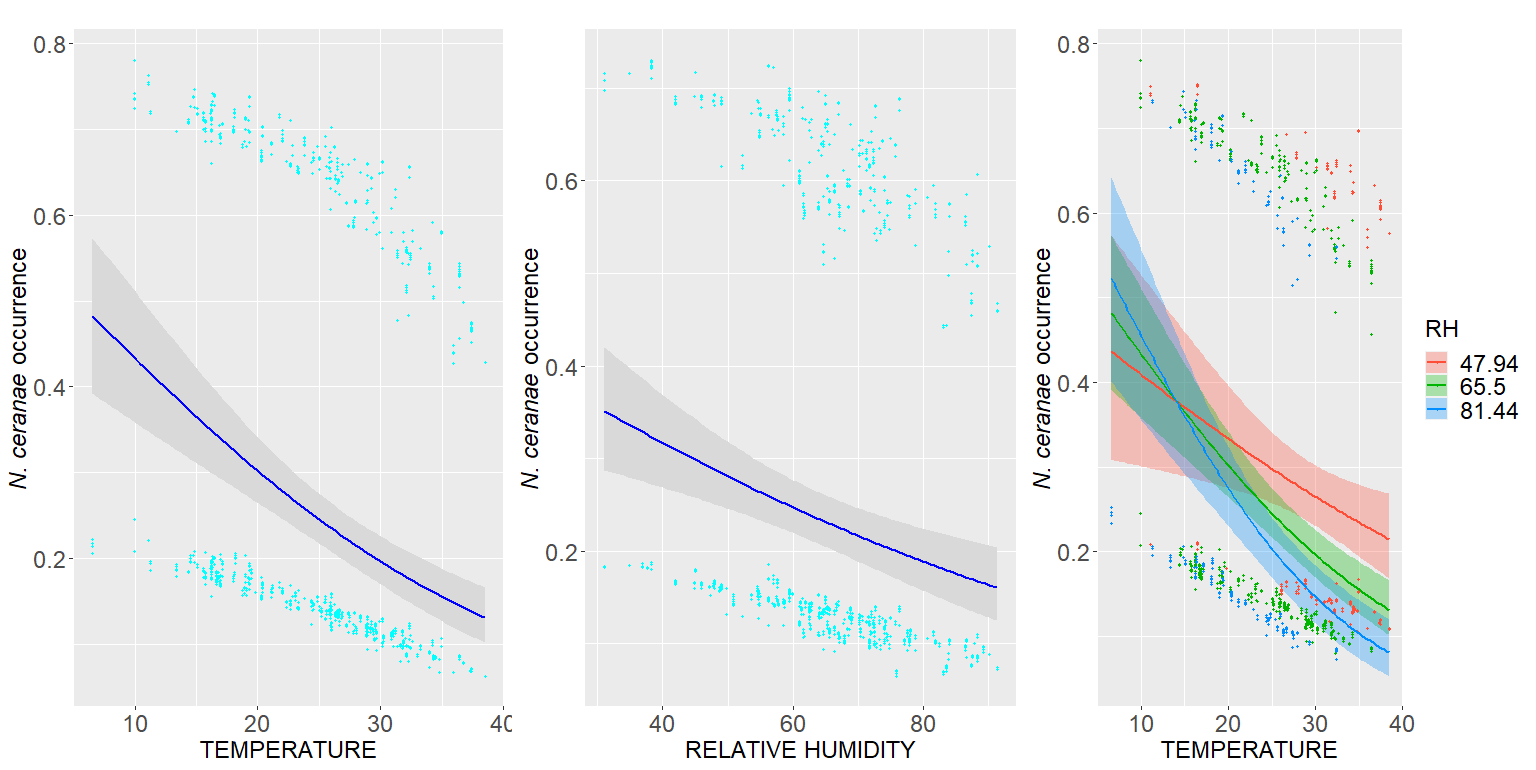


**Figure S2**. Predictors of infection between bees sampled and predictors interactions with GLM_M1_: a) DWV occurrence (Model AIC=4358.6 on 3359 df); b) BQCV occurrence (Model AIC = 3801.8 on 3359 df); c) CBPV occurrence (Model AIC = 2921.4 on 3359 df); d) ABVP occurrence (Model AIC = 2076.7 on 3359 df); e) N. ceranae occurrence (Model AIC = 3566.8 on 3359 df). Shaded colours indicate the 95% confidential interval. RH = relative humidity; ENV.MAN = environmental management. Only graphs of significant variables are represented.

.
