# Supplementary material for: Ecological and social factors influence interspecific pathogens occurrence among bees
Source: Sci Rep. 2024 Mar 1;14:5136. doi: 10.1038/s41598-024-55718-x (PMC10907577; doi:10.1038/s41598-024-55718-x)
Supplement: Supplementary file 12 — Supplementary Figure S3. [file 41598_2024_55718_MOESM12_ESM.docx]

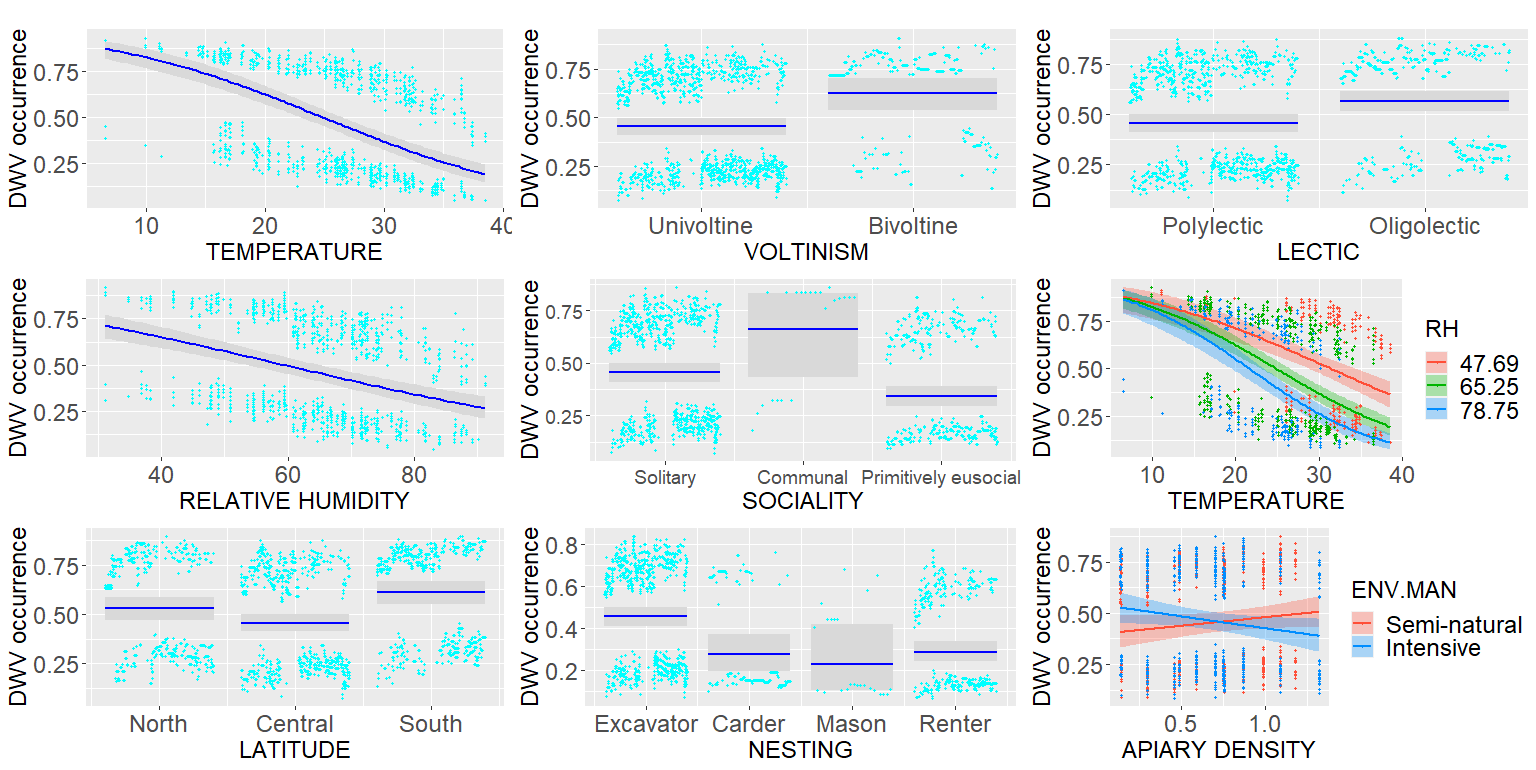


b)

a)


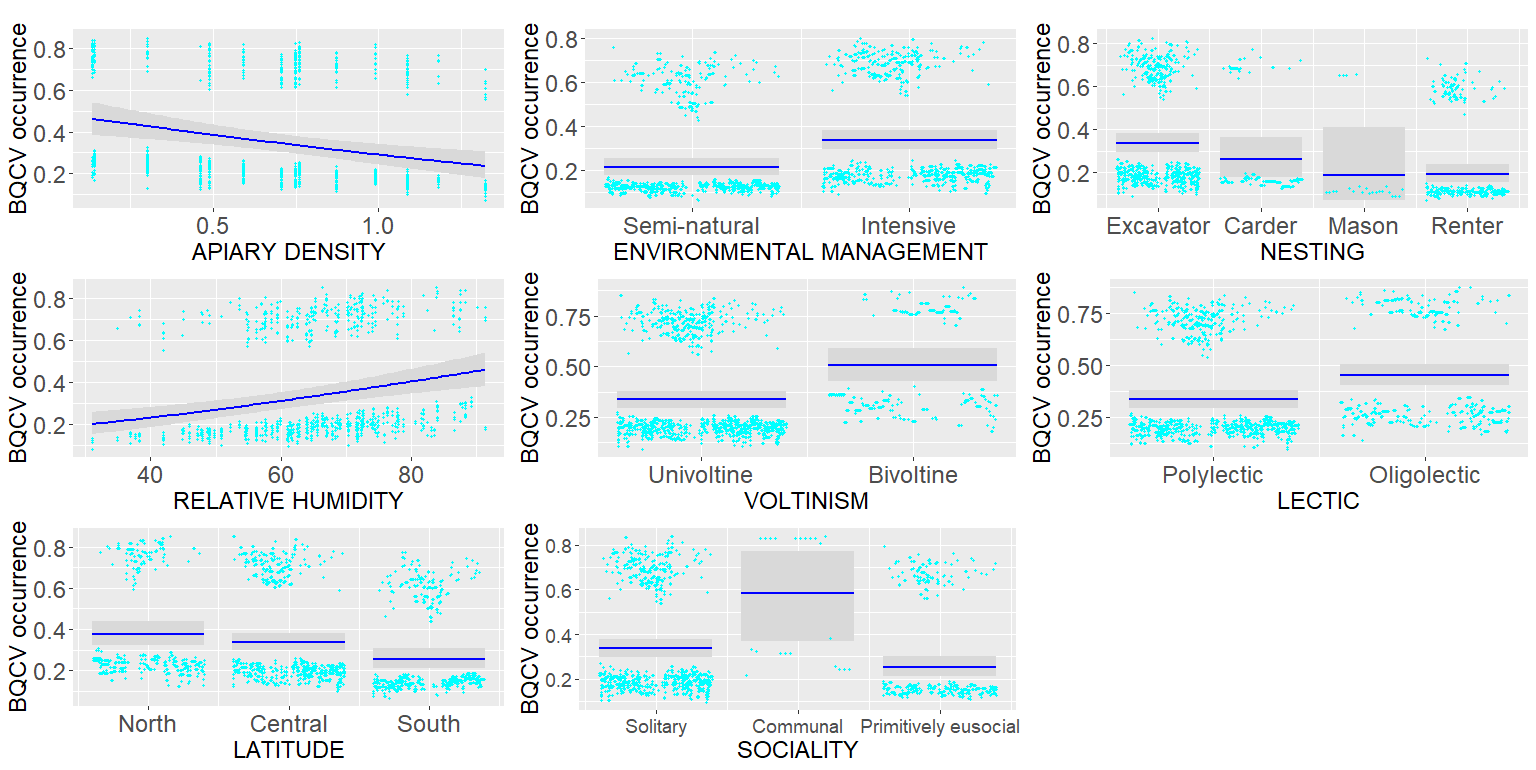


c)


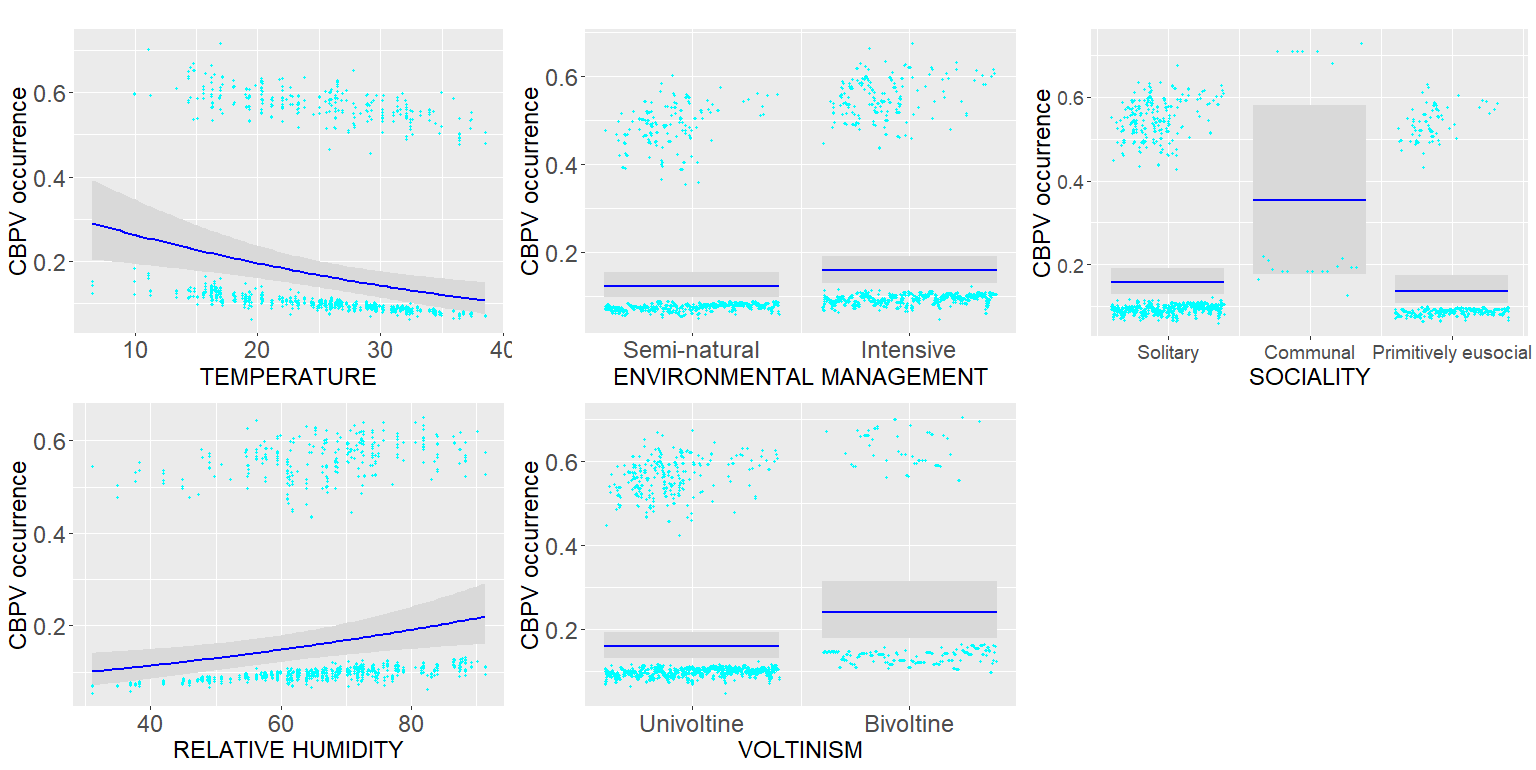


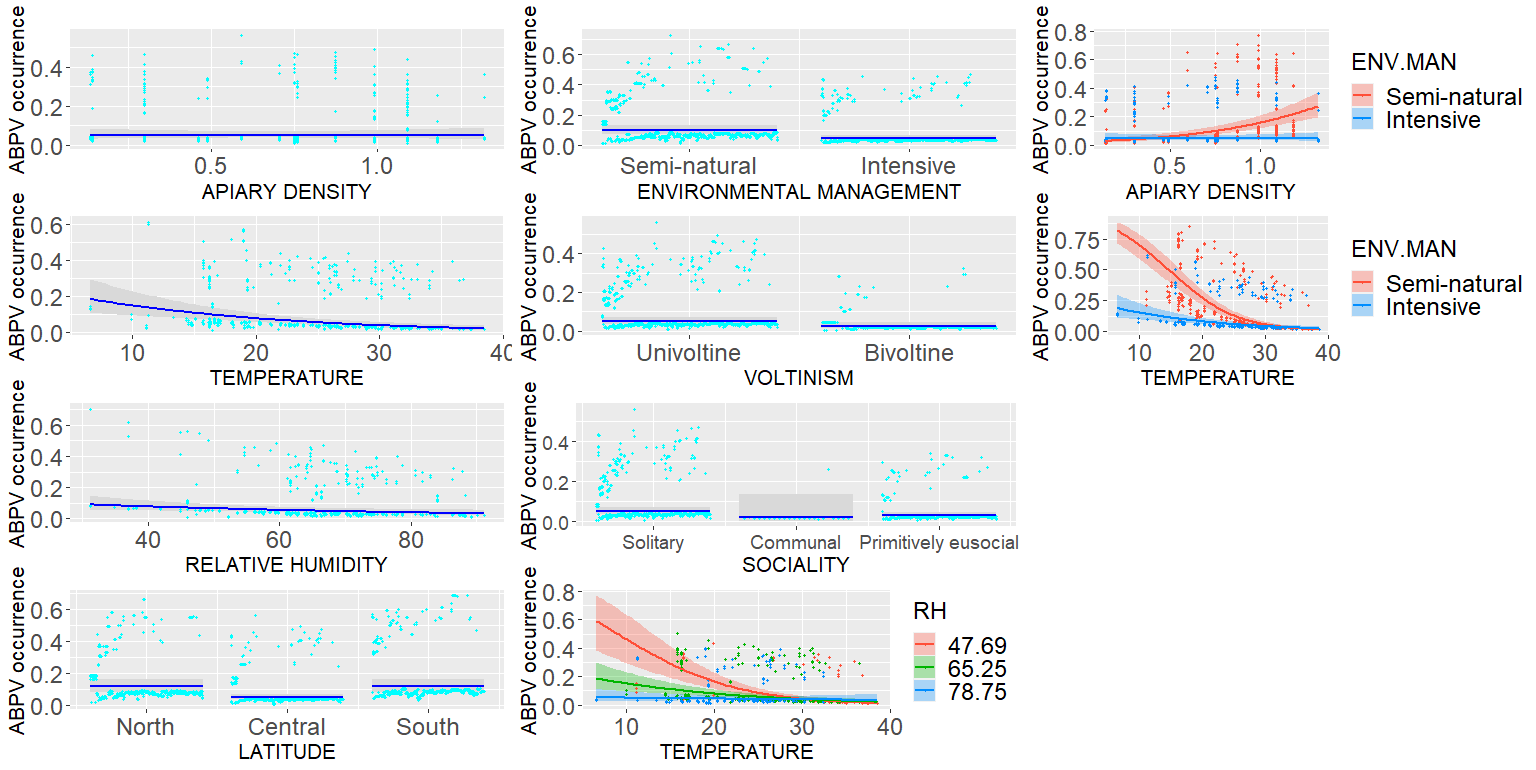


d)

e)


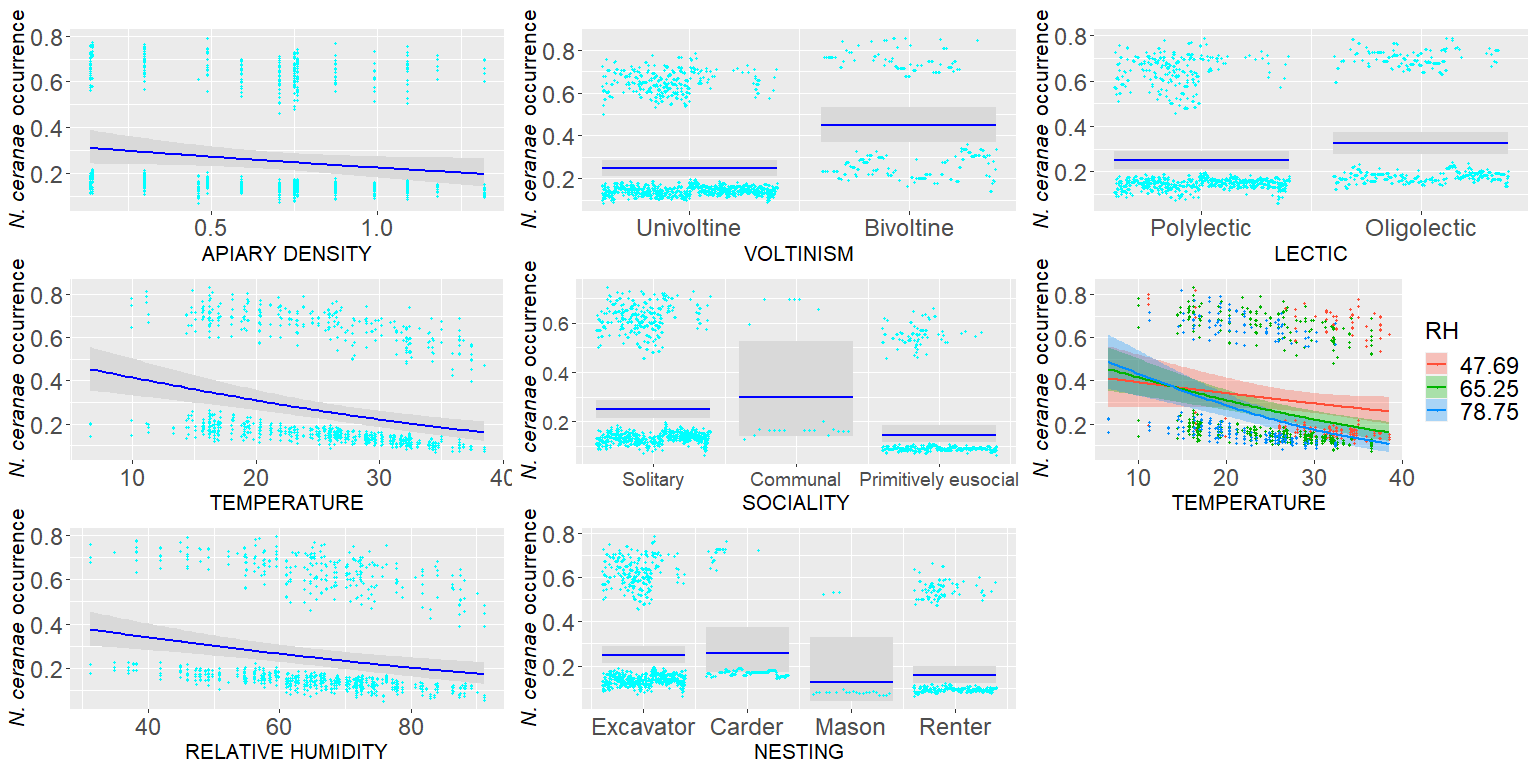


**Figure S3.** Predictors of infection between bees sampled and predictors interactions with GLM_M2_: a) DWV occurrence (Model AIC=3764.4 on 3014 df); b) BQCV occurrence (Model AIC = 3331.9 on 3014 df); c) CBPV occurrence (Model AIC = 2556 on 3014 df); d) ABVP occurrence (Model AIC = 2076.7 on 3359 df); e) N. ceranae occurrence (Model AIC = 3083.3 on 3014 df). Shaded colours indicate the 95% confidential interval. RH = relative humidity; ENV.MAN = environmental management. Only graphs of significant variables are represented.
