# Supplementary material for: Ecological and social factors influence interspecific pathogens occurrence among bees
Source: Sci Rep. 2024 Mar 1;14:5136. doi: 10.1038/s41598-024-55718-x (PMC10907577; doi:10.1038/s41598-024-55718-x)
Supplement: Supplementary file 14 — Supplementary Figure S5. [file 41598_2024_55718_MOESM14_ESM.docx]

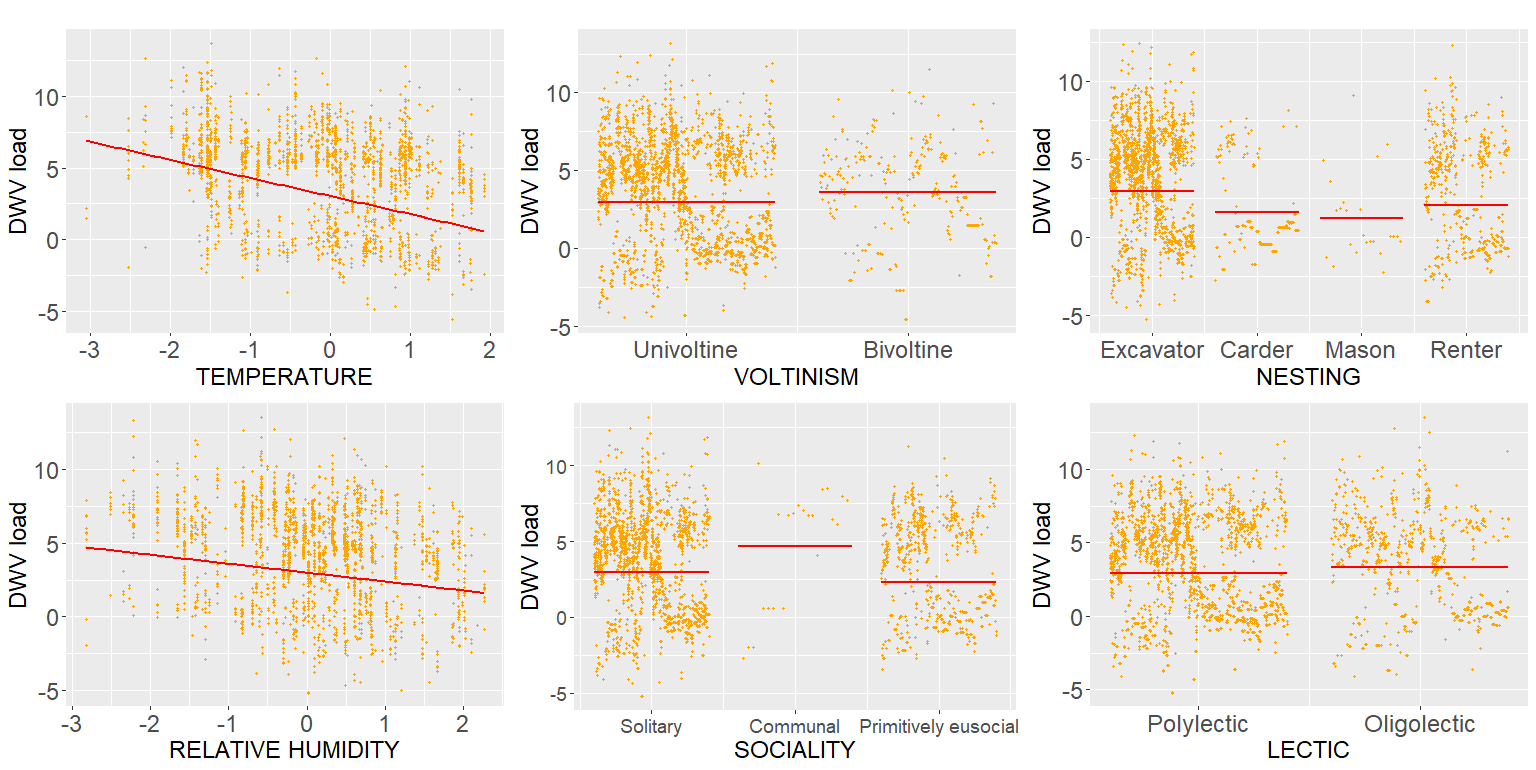


b)

a)


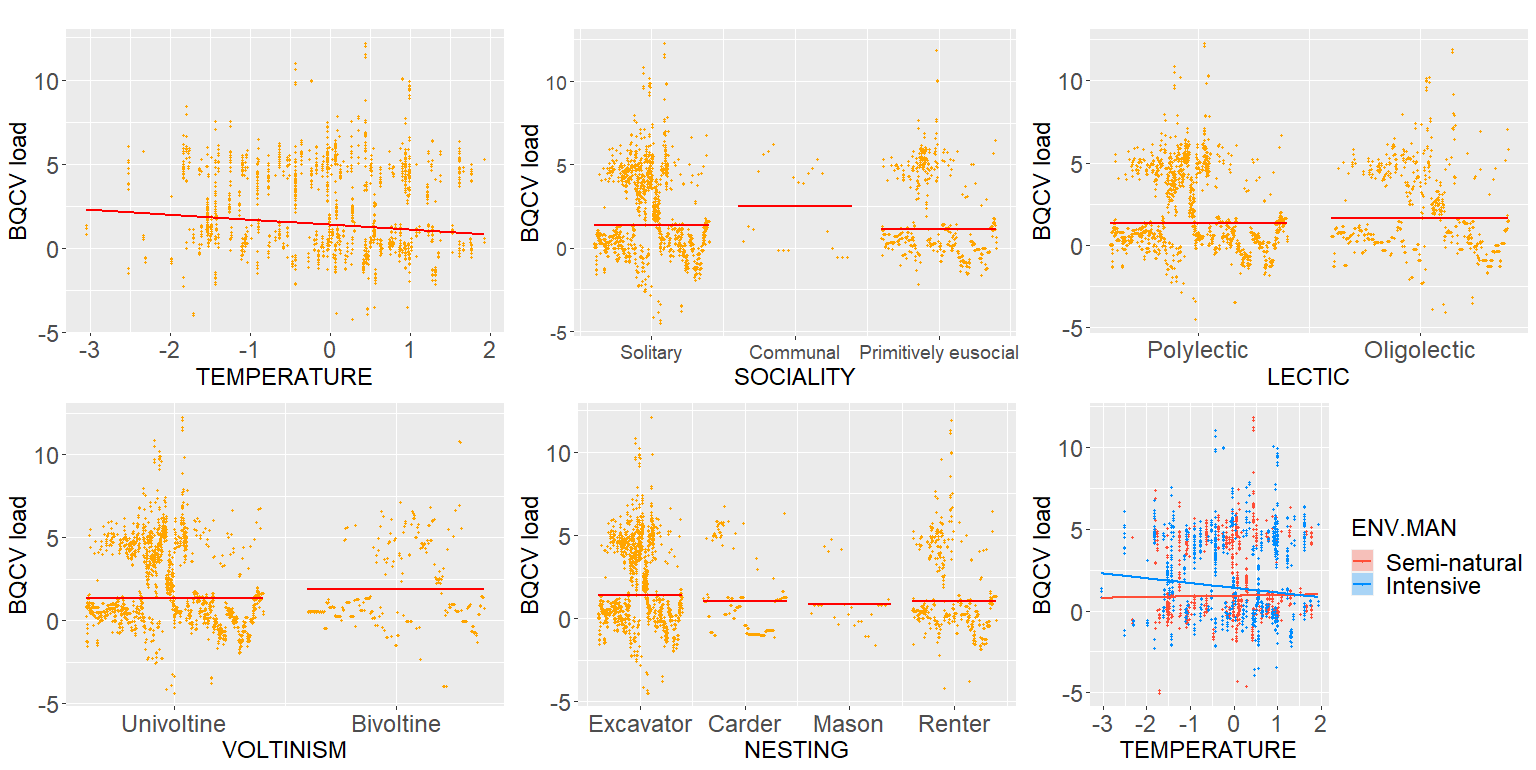


c)


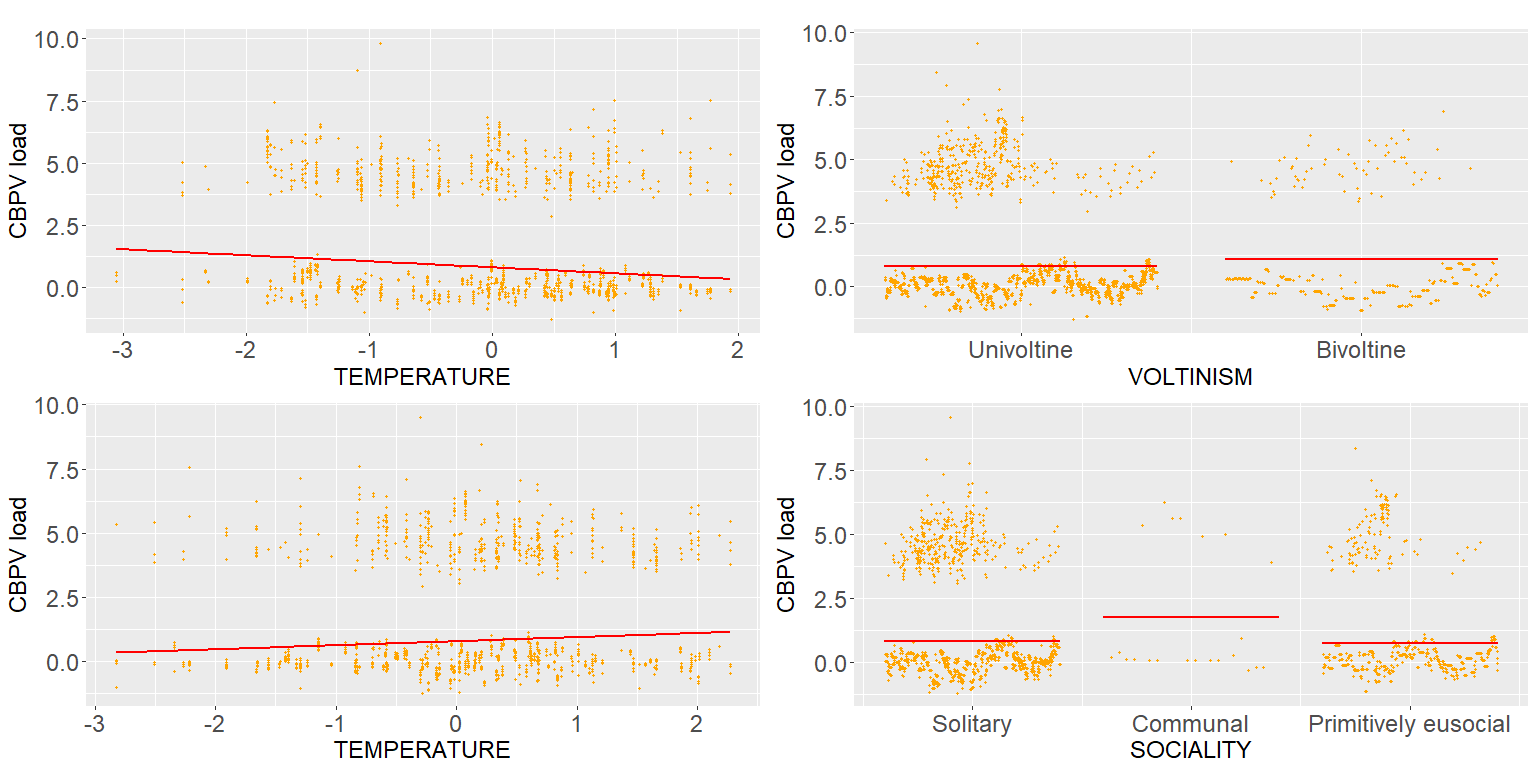


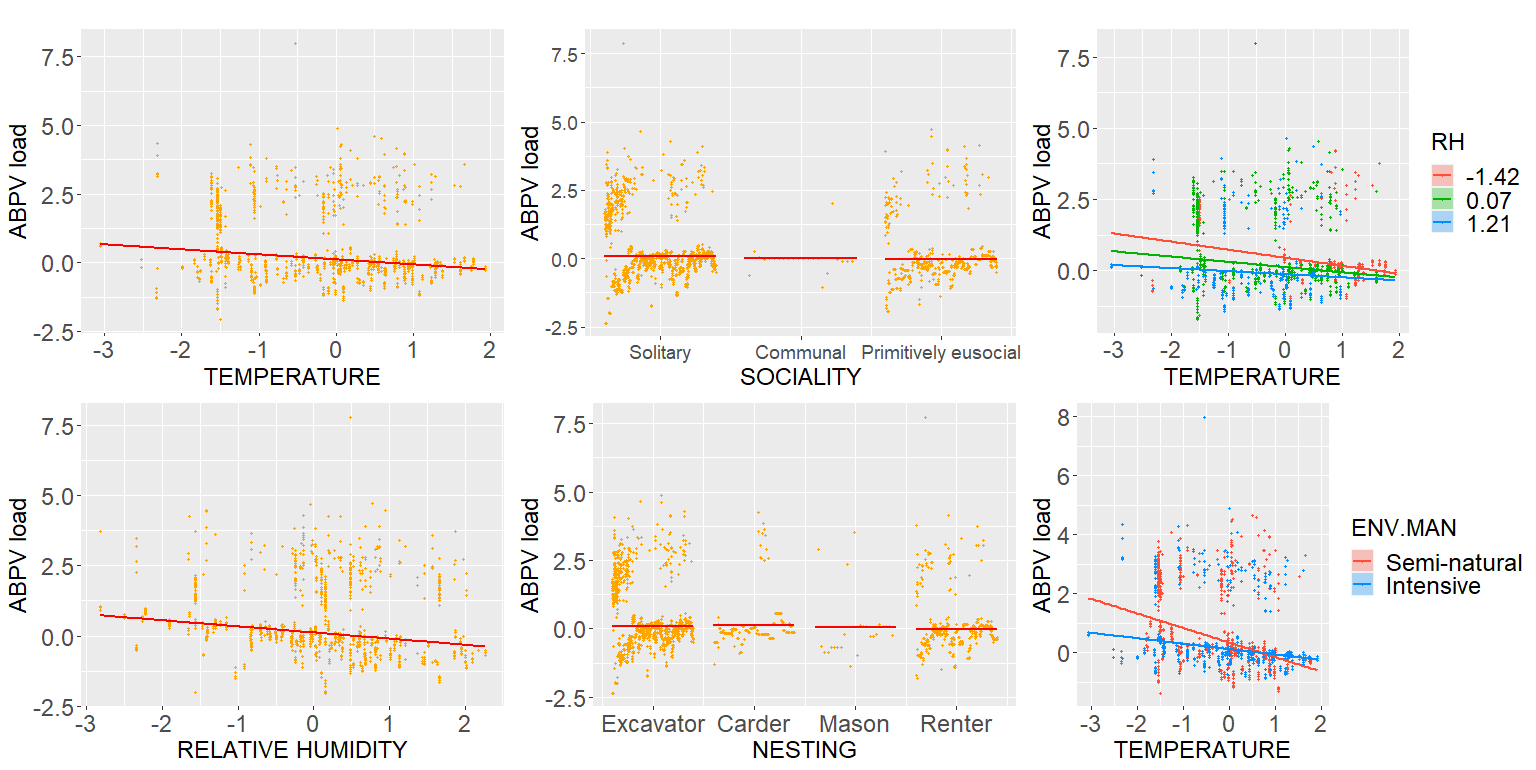


e)

d)


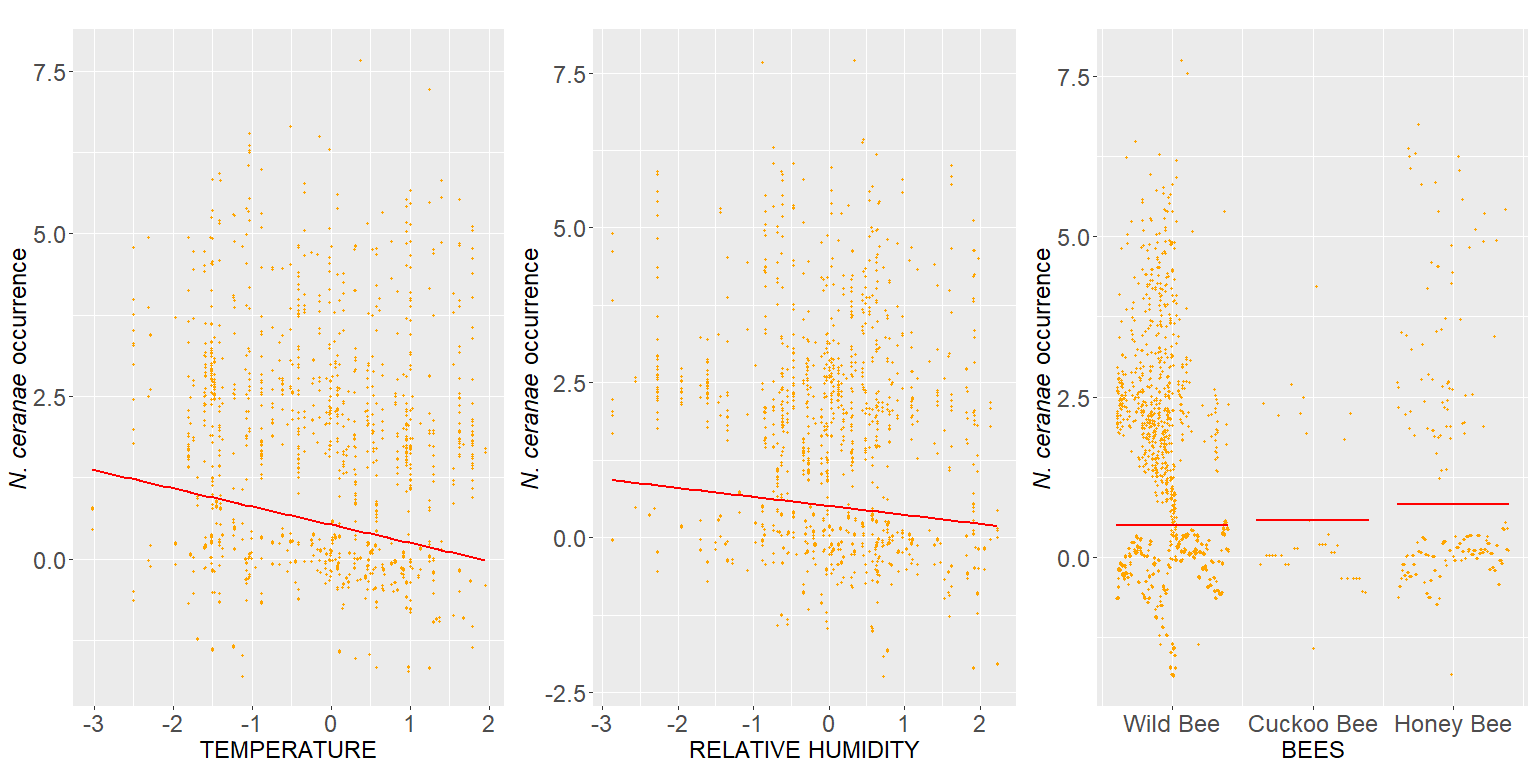


**Figure S5.** Predictors of infection between bees sampled with LMER_M2_: a) DWV load; b) BQCV load; c) CBPV load; d) ABPV load; e). N. ceranae load. Only graphs of significant variables are represented.
